# Effect of oral contraceptive use on cardiorespiratory and immune function in premenopausal women involved in exercise: a systematic review and meta-analysis

**DOI:** 10.3389/fphys.2026.1728032

**Published:** 2026-07-22

**Authors:** Oyesanmi A. Fabunmi, Gugulethu P. Luthuli, Phiwayinkosi V. Dludla, Bongani B. Nkambule

**Affiliations:** 1 Section Sports Medicine, Faculty of Health Sciences, University of Pretoria, Pretoria, South Africa; 2 Health-awareness, Exercise and Cardio-immunologic Research Unit (HECIRU), Department of Physiology, College of Medicine, Ekiti State University, Ado-Ekiti, Ekiti State, Nigeria; 3 Sport Exercise Medicine and Lifestyle Institute (SEMLI), Faculty of Health Sciences, University of Pretoria, Pretoria, South Africa; 4 School of Medicine, College of Health Sciences, University of KwaZulu-Natal, Durban, South Africa; 5 Department of Biochemistry and Microbiology, University of Zululand, KwaDlangezwa, South Africa

**Keywords:** cardiorespiratory function, combined oral contraceptive, exercise, geographical location, immune function, luteal phase, premenopausal women

## Abstract

**Background:**

The beneficial effect of regular physical activity across all bodily functions is well known. The use of oral contraceptives (OCs) is highly prevalent among premenopausal women due to their contraceptive efficacy and favorable safety profile. However, the impact of combined oral contraceptives (COCs) in exercise conditions remains controversial and inconclusive. We aim to explore the effects of COCs on cardiorespiratory and immune function in premenopausal women engaged in exercise when compared with non-COC users.

**Methods:**

Extensive searches of relevant databases, including MEDLINE, CINAHL, Cochrane CENTRAL, and other health sources, were conducted on the EBSCOhost interface from January 2024 to May 2026. All studies that included healthy, physically active premenopausal women and female athletes (15–40 years old) on COC and non-COC users were appraised using the relevant JBI appraisal tools. Data analysis was performed using Review Manager (RevMan) version 5.3. The certainty of evidence was assessed using the Grading of Recommendations, Assessment, Development and Evaluation (GRADE) tool.

**Result:**

The review and meta-analysis included 29 studies comprising 726 participants (recreationally active and trained female athletes) involved in exercise, of whom 390 (53.7%) used COC, and 336 (46.3%) were non-COC users. Overall, 19 studies with a comparator group were included in our meta-analysis. The overall pooled estimates suggest that the use of COC had little to no difference on certain makers of cardiorespiratory and immune function such as heart rate [MD = 0.02, 95% CI (−1.85, 1.9), *p* = 0.98], respiratory parameters [SMD = −0.07, 95% CI (−0.18, 0.04), *p* = 0.28], and IL-6 levels [MD = -0.88, 95% CI (2.75, 0.98), *p* = 0.35)] when compared with the non-COC group. Similarly, there is little to no difference in the body composition (BMI) [MD = -0.21, 95% CI (-0.59, 0.18), *p* = 0.3)] and metabolic changes [SMD = -0.15, 95% CI (-0.43, 0.12), *p* = 0.36)] among COC users compared with the non-COC group. Nonetheless, the certainty of evidence across all measured outcomes in the included studies is low.

**Conclusion:**

The current evidence does not demonstrate a consistent detrimental effect of COC use on cardiorespiratory or immune outcomes among physically active premenopausal women; however, confidence in these findings remains limited due to the low certainty of the available evidence. The low certainty of the evidence across the included studies is attributable to small sample sizes, high risk of bias, varied methodological approaches, and a lack of data from specific geographical locations, among other factors. Thus, caution should be exercised when extrapolating these findings for consultation or decision-making regarding COC use in and out of clinical settings.

**Systematic Review Registration:**

https://www.crd.york.ac.uk/PROSPERO/view/CRD42021265257, identifier CRD42021265257.

## Highlights

- The use of COC is not adversely detrimental to the cardiorespiratory and immune function and other health-related indicators among recreationally active premenopausal women and trained athletes.
- Available evidence suggests little to no difference in most cardiorespiratory and immune outcomes between COC users and non-users, although the certainty of evidence remains low.

## 1 Introduction

The growing popularity of oral contraceptives (OCs) containing synthetic or exogenous versions of ethinyl estradiol and a generation of progestin (COC) or progestin-only has contributed to the high prevalence of use in premenopausal women across different geographic regions (UN. Population Division, 2019). Aside from the purpose of birth control, physically active premenopausal women and female athletes adopt the use of COC for cycle control during training and competition and to mitigate certain conditions involving menstrual disorders, endometriosis, and polycystic ovarian syndrome (Martin et al., 2018; Palacios et al., 2019; Elliott-Sale et al., 2020; Burchardt et al., 2021; Steinberg Weiss et al., 2021). Monophasic COC pills have the same amount of hormones, unlike other forms of COC, such as the biphasic and triphasic ones, which contain varied amounts of hormones (Van Vliet et al., 2011). Despite the perceived benefits, the risk of some adverse outcomes, such as arterial and venous thrombotic events, as well as depressive symptoms, remains a concern among women exposed to COC (Oedingen et al., 2018; De Wit et al., 2020; Clarke et al., 2021).

Emerging evidence regarding the impact of COC on cardiorespiratory function (CRF) and exercise performance remains inconsistent. Briefly, CRF is an integrative association involving respiratory and cardiovascular responses to meet an individual’s metabolic demands, which are fundamental components of health and well-being (Delaney and Barker). A comprehensive assessment of this integrative process provides a predictive overview of CRF and exercise performance in diverse population settings (Rubin et al., 2009; Ellis et al., 2024). The inconsistency regarding COC and its impact on CRF is linked to variations in ethinylestradiol and progestin doses, modality of exercise, and other factors (Van Den Heuvel et al., 2005; Elliott-Sale et al., 2013; Thompson et al., 2020).

Previous evidence showed that the use of OC pills results in slightly inferior exercise performance, on average, when compared to naturally menstruating women, where the group-level effect was reportedly trivial (Elliott-Sale et al., 2020). In contrast, other studies reported no significant effects of COC or the normal menstrual phase on exercise performance among participants (Thompson et al., 2020; Thompson et al., 2021). The interpretation of findings associating COC use with adverse effects on the index of CRF and exercise performance in young women is constrained by methodological limitations, including small sample sizes in most studies and variations in COC formulation, among other factors (Elliott-Sale et al., 2021; Schumpf et al., 2023).

One notable index of CRF is maximal oxygen consumption (VO_2_ max), among others. Emerging evidence suggests an association between COC use and changes in maximal oxygen consumption (VO_2_ max) in certain individuals (Kaminsky et al., 2015; Elliott-Sale et al., 2020; Dun et al., 2022). It is worth noting that VO_2_ max in exercise conditions reflects changes in local metabolic demand and is primarily linked to changes in cardiac output and arterio-venous oxygen difference as well as neural pathways and central integration in the higher centers (Saltin et al., 2006; Fukuba et al., 2007; Levine, 2008; Wagner, 2015). Potential mechanisms by which COC may alter CRF include modulation of vascular tone, mitochondrial function, plasma volume, and tissue oxygenation. In the luteal phase of the menstrual cycle, increased estrogen levels enhance NO availability and vasodilatation and increase mitochondrial biogenesis and oxygen delivery at the tissue level (Stirone et al., 2005; Stathokostas et al., 2009) while reducing sympathetic activity and vasoconstriction (Carter et al., 2013). This process contributes to the acute inflammatory response during exercise. Progesterone has also been shown to have anti-atherosclerotic effects (Okamoto et al., 2017), facilitate fluid retention, and increase hemoglobin level, contributing to increased tissue oxygenation (Mullen et al., 2020; Badenhorst et al., 2021). Previous evidence suggests a 11% reduction in VO_2_ max in women who used COC for 4 months compared with baseline (Casazza et al., 2002). In contrast, emerging evidence showed no significant differences in VO_2_ max or other cardiovascular parameters, such as heart rate, stroke volume, and cardiac output, between monophasic COC users and non-users (Gordon et al., 2018). It remains inconclusive whether the prolonged use of different COC formulations also affects other measures of CRF, particularly when accounting for variations in exercise type and intensity (Schumpf et al., 2023). Different generations of progestogens used in combined oral contraceptives differ in their chemical structure, androgenic activity, metabolic effects, and cardiovascular risk profile, with newer generations generally designed to minimize androgenic side effects and improve tolerability while maintaining contraceptive efficacy. First- and second-generation progestins, such as norethindrone and levonorgestrel, tend to exhibit greater androgenic activity, whereas third- and fourth-generation progestins, including desogestrel, norgestimate, and drospirenone, are associated with lower or anti-androgenic effects (Louw-du Toit et al., 2017).

Moreover, evidence regarding the effects of COCs on inflammatory mediators and vascular function during exercise remains limited and inconsistent (Timmons et al., 2005; Cauci et al., 2017; Larsen et al., 2020; Notbohm et al., 2023b). Emerging evidence suggests that COC use is associated with an increased release of inflammatory cytokines in women of reproductive age, which may have broader implications for CF (Piltonen et al., 2012; Cauci et al., 2021; Eagan et al., 2021). In exercise conditions, inflammatory myokines such as interleukin (IL)-6, IL-1ra, and IL-10 are released from the skeletal muscle. They play a role in muscle repair and morphological adaptations and inhibit pro-inflammatory cytokines such as tumor necrosis factor-alpha (TNF-α), among others (Deng et al., 2012; Zhang et al., 2013; Chazaud, 2016; Welc et al., 2020). Systemic circulation of several other myokines is also linked to vascular adaptation, in which their anti-inflammatory effects stimulate nitric oxide (NO) release, mitigating oxidative stress and arterial stiffness and thereby increasing oxygen delivery to various tissues (Steensberg et al., 2007). Depending on the exercise intensity, duration, and familiarity, these physiological responses may shift toward a pathological state (Petersen and Pedersen, 2005; Gleeson, 2007; Gleeson et al., 2011; Cerqueira et al., 2020; Pranoto et al., 2023). Although empirical data indicate an inverse correlation between specific cardiorespiratory and immunological markers during physical exertion, the precise extent to which combined oral contraceptives (COCs) modulate these fluctuations across distinct menstrual phases and environmental exercise profiles remains unresolved (Church et al., 2002; Soltani et al., 2020; Payandeh et al., 2022). Looking at the physiological mechanisms by which exogenous sex hormones influence cardiorespiratory performance, such as increased vascular responsiveness, oxygen transport capacity, autonomic regulation, acute inflammatory response, and substrate utilization during exercise, we hypothesized that COC use could alter selected markers of cardiorespiratory and immune function when compared with naturally menstruating women.

It is noteworthy that previous reviews have assessed the impact of OC use on exercise performance; however, such studies have not taken into account the implications of immune function and other critical indices that are valuable during the comprehensive assessment of CRF in women of reproductive age (Elliott-Sale et al., 2020; Schumpf et al., 2023). Hence, our aim is to provide a comprehensive synthesis of the available evidence on the effects of COC on cardiorespiratory and immune function in premenopausal women engaged in exercise compared with non-COC users. Such analysis is consistent with determining the potential role of different COC formulations and exposure duration in exercise conditions. Given the role of available COC formulations on cardiorespiratory and immune function, it may help provide insight and guidance for the well-being of recreationally active young women and female athletes who need contraceptive hormones in their daily lives.

## 2 Methods

This systematic review and meta-analysis was reported according to the preferred reporting items for systematic reviews and meta-analysis (PRISMA) guidelines (Page et al., 2021). This systematic review was registered with the International Prospective Register of Systematic Reviews (PROSPERO: CRD42021265257), and the protocol was published (Fabunmi et al., 2024). Thus, a comprehensive and systematic search of published studies was conducted to address the following review question:

1. What is the effect of COC intake on markers of cardio-respiratory and immune function in premenopausal women engaged in exercise compared to the control groups not on COC?

### 2.1 Eligibility criteria

The study types included cross-sectional, cohort, and randomized control trials (RCTs). Our study included healthy premenopausal women (recreationally active individuals and trained female athletes) aged 15–40 years who engage in exercise and use COC. The rationale for the age limit of 40 years is to limit the confounding influence of perimenopausal status. The comparators are physically active, naturally menstruating individuals (recreationally active individuals and trained female athletes), and those in the inactive pill phase. The inactive pill phase stands as the period when an individual withdraws from COC usage. Studies were eligible if they included an independent comparator group of naturally menstruating women or used within-subject comparisons across active and inactive pill phases. The exercise conditions are aerobic and anaerobic exercise at low, moderate, and high intensities, within the average frequency recommended by the World Health Organization (WHO) guidelines on physical activity and exercise (Bull et al., 2020). Studies reporting on smokers were excluded from our review because smoking is regarded as an unhealthy lifestyle, which contradicts the focus of our study regarding healthy participants. Only original research was included, and the bibliographies of all included and excluded studies were searched for relevant citations without language restriction.

### 2.2 Outcomes

The primary outcomes of the systematic review include evaluating the markers of cardiorespiratory function such as submaximal oxygen consumption (VO_2_), maximal oxygen consumption (VO_2_ max), carbon dioxide output (VCO_2_), minute ventilation (VE), respiratory exchange ratio (RER), and heart rate (HR) (Zebrowska et al., 2018) as well as acute phase reactants such as tumor necrosis factor (TNF-α), C-reactive protein (CRP), interleukin (IL)-6, IL-1β, IL-8, and IL-10, which are considered markers of immune function (Beavers et al., 2010; Moganti et al., 2017). The secondary outcomes included other health-related outcomes, such as body composition (body mass index [BMI]) and metabolic changes (lipid profiles, blood glucose, or blood lactate levels). The influence of exercise on these outcomes contributes to an individual’s cardiorespiratory and immune function.

### 2.3 Search strategy and information sources

The search strategy includes the text words contained in the titles and abstracts of relevant articles as well as the index terms used to describe the articles, such as contraceptives, cardio-respiratory functions, immune activation, and premenopausal women (**Supplementary Table 1**). The keywords and MeSH terms used included “oral contraceptive pills”, “birth control pills’’, “oral contraceptives”, “contraceptives”, “combined oral contraceptives”, “exercise”, “physical activity”, “cardiorespiratory fitness”, “immune activation”, “immune function”, “immunity”, “premenopausal women”, and “adult females”. We conducted a comprehensive search of EBSCOhost interface to access the following databases: MEDLINE, Cumulative Index to Nursing and Allied Health Literature (CINAHL), Cochrane Central Register of Clinical Trials (CENTRAL), and other relevant health sources. We also searched OpenGrey (System Information on Grey Literature in Europe) (www.opengrey.eu) and ClinicalTrials.gov to augment relevant sources of information. In addition, the reference lists of the selected studies were scanned, and forward citation tracking in Google Scholar was conducted to identify relevant studies. The initial search was conducted between January and October 2024 and updated in May 2026. The search strategy was adapted for each database as needed.

### 2.4 Study selection

Two independent authors conducted the screening of studies to be included in our review to avoid inconsistency regarding the eligibility of studies. Firstly, the title and abstracts were screened, and the full texts of eligible studies were retrieved for further screening. The Mendeley desktop reference manager (version 1.19.4) was used to retrieve and archive relevant studies as well as remove study duplicates. When disagreements ensued, the third author was consulted for arbitration.

### 2.5 Data items and collection process

Firstly, two reviewers independently piloted the data extraction process with a data extraction sheet involving three of the included studies. The third reviewer resolved disagreements between the authors where necessary. The data extraction sheet was used to collect all relevant information on essential reporting items such as the name of the author/s, year of study publication, country where the study was conducted, main findings, study design, type of OC used, dosage, duration of use, types of exercise, intensity, population (sample size), and age of participants.

### 2.6 Methodological quality assessment of the included studies

Two independent authors (OAF and GPL) assessed the methodological quality of all the included studies based on the study design using the relevant standardized appraisal checklist from the Joanna Briggs Institute (JBI) (Tufanaru et al., 2020). The JBI checklist offers a specific appraisal for each study design. When disagreements ensued, a third author (BBN) abridged the process. An outline of the results of the detailed analysis was created, and the quality score of all included studies was graded and scored as “low” (one to four points), “moderate” (five to eight points), and “high” (>9 points). The levels of the inter-rater agreement were assessed using Cohen’s kappa (Downs and Black, 1998) where a score ≤0 indicates no agreement, 0.01–0.20 is none to slight, 0.21–0.40 is fair, 0.41–0.60 is moderate, 0.61–0.80 is substantial, and 0.81–1.00 is an almost perfect agreement (McHugh, 2012) (**Supplementary Table 2**).

### 2.7 Data synthesis and statistical analysis

Higgin’s *I*^2^ statistic was used to assess statistical heterogeneity. In cases of substantial heterogeneity (*I*^2^ > 50%), a random-effects model was used to generate pooled effect estimates (Schroll et al., 2011). Outcomes with the same effect estimates were reported as the mean difference (MD), while different effect estimates were reported as standardized mean difference (SMD) and 95% confidence interval (CI). The effect size of our estimated outcome is small (0.2–0.49), moderate (0.5–0.79), or large (≥0.8). Where necessary, we performed data transformation to standard deviation (SD), where CI was reported in some studies (Higgins et al., 2019). Data analysis was performed using Review Manager (RevMan) version 5.3. Publication bias was assessed via the visual inspection of funnel plots, where a perfect symmetry indicates no publication bias (**Supplementary Figures 1A–D**). A *p*-value <0.05 was considered statistically significant.

### 2.8 Certainty of evidence

The quality of evidence was evaluated using the Grading of Recommendations, Assessment, Development, and Evaluation (GRADE) tool (Ryan and Hill, 2019). The findings were presented in the summary of findings (SoF) table (**Supplementary Table 3**).

## 3 Results

### 3.1 Literature search and characteristics of the included studies

**Figure 1:**
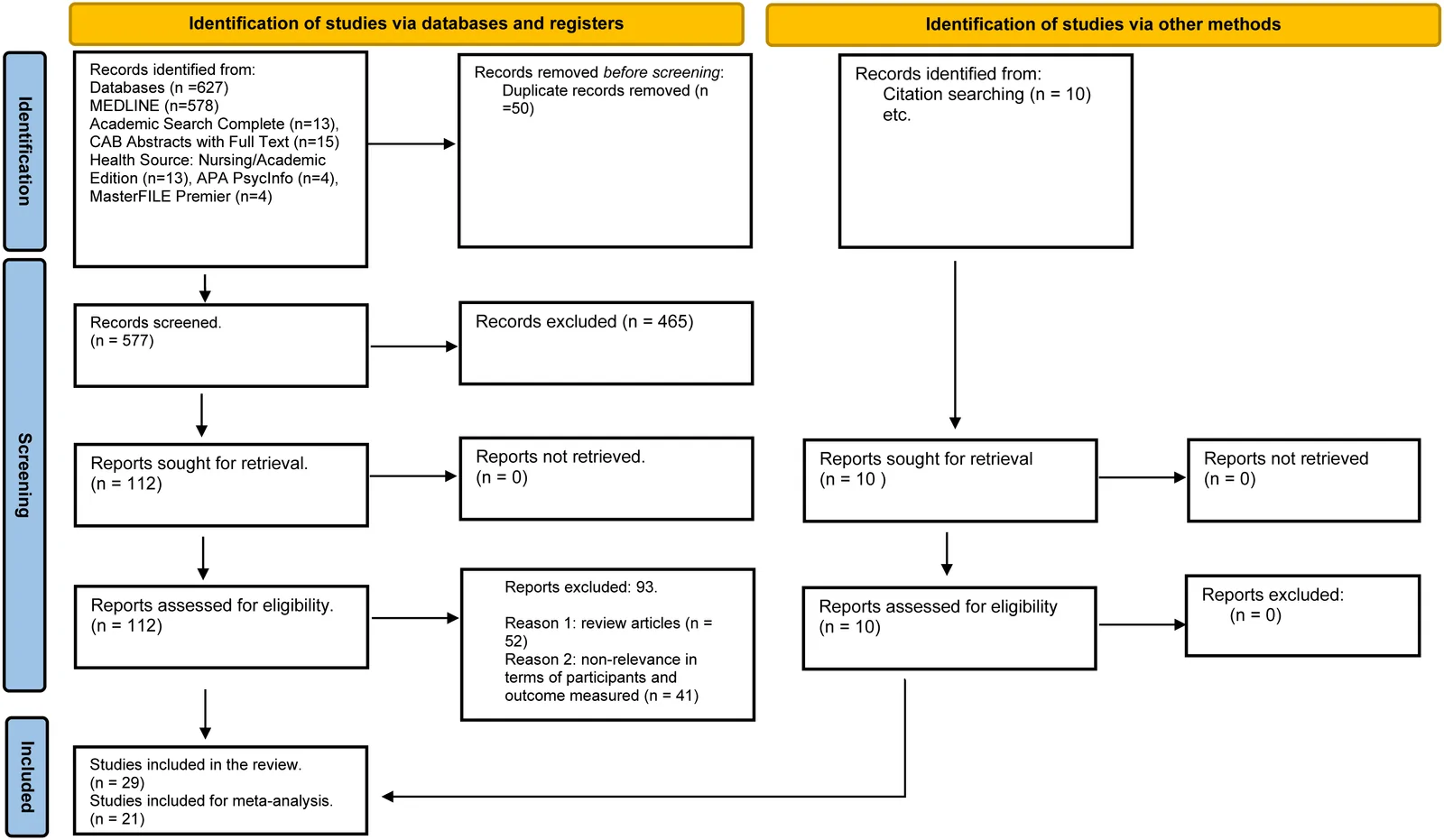
PRISMA study identification and selection flow chart.

The full results of the search strategy and study selection process are presented in the PRISMA flow diagram (Page et al., 2021) (**Figure 1**). As outlined in **Figure 1**, a total of 627 studies were identified and retrieved using the predetermined search strategy and screened for eligibility. In the end, 598 studies were excluded. Among the excluded studies, 42 were reviews and 556 were irrelevant to the topic of interest. More so, all included studies were published between 1986 and 2023. Overall, 29 studies met the inclusion criteria and were included in the review.

The total population across all studies comprises 726 participants, of whom 390 (53.7%) were taking COC and 336 (46.3%) were non-COC. The median age of the participants in the included studies was 23 years. Among the included studies, 22 reported on recreationally active and highly trained females (Umlauff et al., 2021; Notelovitz et al., 1987; Bryner, 1996; Lynch and Nimmo, 1998; Giacomoni and Falgairette, 2000; Lynch et al., 2001; Casazza et al., 2002; Redman et al., 2005; Timmons et al., 2005; Santos et al., 2008; Rebelo et al., 2010; Vaiksaar et al., 2011a; Joyce et al., 2013; Isacco et al., 2015; Schaumberg et al., 2017; Gordon et al., 2018; Ihalainen et al., 2019; Mattu et al., 2020; Schaumberg et al., 2020; Taipale-Mikkonen et al., 2021; Gomes et al., 2022; Notbohm et al., 2023b), six reported on trained female athletes (Lebrun et al., 2003; Procter-Gray et al., 2008; Rechichi et al., 2008; Larsen et al., 2020; Bozzini et al., 2021; Nakamura and Nose-Ogura, 2021), and one reported on both (Vaiksaar et al., 2011b). Moreover, 26 studies reported on exercise equipment such as cycle ergometer, rowing ergometer, treadmill, contact mat, Smith machine, and T-FORCE system (Umlauff et al., 2021; Notelovitz et al., 1987; Bryner, 1996; Lynch and Nimmo, 1998; Giacomoni and Falgairette, 2000; Lynch et al., 2001; Casazza et al., 2002; Lebrun et al., 2003; Redman et al., 2005; Timmons et al., 2005; Rechichi et al., 2008; Santos et al., 2008; Rebelo et al., 2010; Vaiksaar et al., 2011a; Vaiksaar et al., 2011b; Joyce et al., 2013; Isacco et al., 2015; Schaumberg et al., 2017; Gordon et al., 2018; Mattu et al., 2020; Schaumberg et al., 2020; Bozzini et al., 2021; Nakamura and Nose-Ogura, 2021; Taipale-Mikkonen et al., 2021; Gomes et al., 2022; Notbohm et al., 2023b), while in three studies none was reported (Procter-Gray et al., 2008; Ihalainen et al., 2019; Larsen et al., 2020).

**Figure 2:**
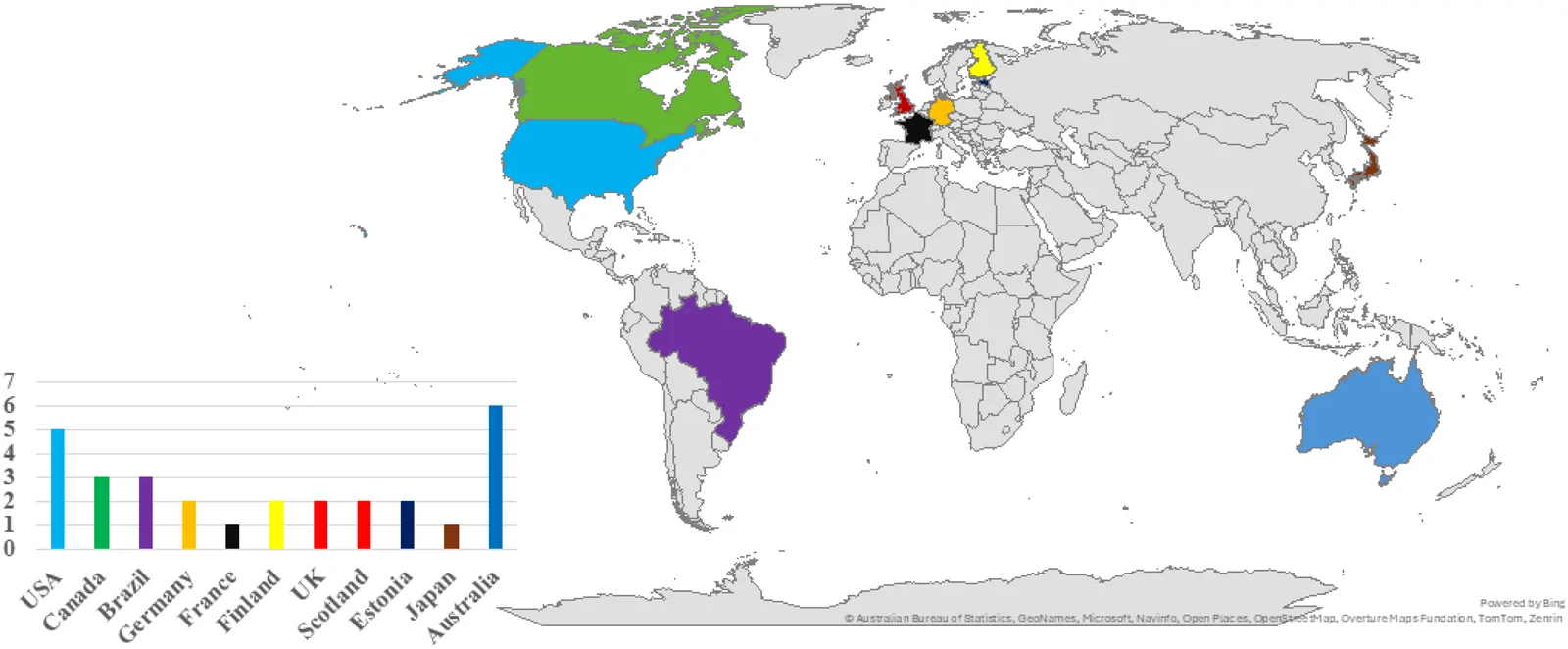
Geographical distribution of all the included studies.

The study design consisted of 11 cross-sectional studies (Notelovitz et al., 1987; Santos et al., 2008; Rebelo et al., 2010; Schaumberg et al., 2017; Ihalainen et al., 2019; Larsen et al., 2020; Mattu et al., 2020; Schaumberg et al., 2020; Bozzini et al., 2021; Nakamura and Nose-Ogura, 2021; Gomes et al., 2022), four RCTs (Giacomoni and Falgairette, 2000; Lebrun et al., 2003; Redman et al., 2005; Procter-Gray et al., 2008), and 14 cohort studies (Umlauff et al., 2021; Bryner, 1996; Lynch and Nimmo, 1998; Lynch et al., 2001; Casazza et al., 2002; Timmons et al., 2005; Rechichi et al., 2008; Vaiksaar et al., 2011b; Vaiksaar et al., 2011a; Joyce et al., 2013; Isacco et al., 2015; Gordon et al., 2018; Taipale-Mikkonen et al., 2021; Notbohm et al., 2023b). Across the studies, only 21 with an independent comparator group (non-COC) were included in the meta-analysis (Delaney and Barker; Van Den Heuvel et al., 2005; Rubin et al., 2009; Van Vliet et al., 2011; Elliott-Sale et al., 2013; Kaminsky et al., 2015; Martin et al., 2018; Oedingen et al., 2018; Palacios et al., 2019; UN. Population Division, 2019; De Wit et al., 2020; Elliott-Sale et al., 2020; Thompson et al., 2020; Burchardt et al., 2021; Clarke et al., 2021; Elliott-Sale et al., 2021; Steinberg Weiss et al., 2021; Thompson et al., 2021; Dun et al., 2022; Schumpf et al., 2023; Ellis et al., 2024), while the remaining 10 lacked such a group (Van Vliet et al., 2011; Martin et al., 2018; Palacios et al., 2019; UN. Population Division, 2019; De Wit et al., 2020; Elliott-Sale et al., 2020; Burchardt et al., 2021; Steinberg Weiss et al., 2021). The geographical distribution of the included studies comprised 11 from Europe (Umlauff et al., 2021; Lynch and Nimmo, 1998; Giacomoni and Falgairette, 2000; Lynch et al., 2001; Vaiksaar et al., 2011b; Vaiksaar et al., 2011a; Isacco et al., 2015; Gordon et al., 2018; Ihalainen et al., 2019; Taipale-Mikkonen et al., 2021; Notbohm et al., 2023b), eight from North America (Notelovitz et al., 1987; Bryner, 1996; Casazza et al., 2002; Lebrun et al., 2003; Timmons et al., 2005; Procter-Gray et al., 2008; Mattu et al., 2020; Bozzini et al., 2021), three from South America (Santos et al., 2008; Rebelo et al., 2010; Gomes et al., 2022), six from Australia (Redman et al., 2005; Rechichi et al., 2008; Joyce et al., 2013; Schaumberg et al., 2017; Larsen et al., 2020; Schaumberg et al., 2020), and one from Asia (Nakamura and Nose-Ogura, 2021) (**Figure 2**).

We also assessed the formulation and generational-type of COC in the included studies. Briefly, 25 studies reported on monophasic COC formulations (Umlauff et al., 2021; Notelovitz et al., 1987; Bryner, 1996; Lynch and Nimmo, 1998; Giacomoni and Falgairette, 2000; Lynch et al., 2001; Redman et al., 2005; Procter-Gray et al., 2008; Rechichi et al., 2008; Santos et al., 2008; Rebelo et al., 2010; Vaiksaar et al., 2011a; Vaiksaar et al., 2011b; Joyce et al., 2013; Isacco et al., 2015; Schaumberg et al., 2017; Gordon et al., 2018; Ihalainen et al., 2019; Larsen et al., 2020; Mattu et al., 2020; Schaumberg et al., 2020; Nakamura and Nose-Ogura, 2021; Taipale-Mikkonen et al., 2021; Gomes et al., 2022; Notbohm et al., 2023b), while three studies reported on triphasic COC formulations (Casazza et al., 2002; Lebrun et al., 2003; Timmons et al., 2005), and only one study did not indicate the formulation of the COC used (Bozzini et al., 2021). Furthermore, the formulations of COCs reported in all included studies belong to the first, second, third, and fourth generations. In terms of treatment duration of exposure to oral contraceptives, 13 studies (Notelovitz et al., 1987; Bryner, 1996; Casazza et al., 2002; Lebrun et al., 2003; Redman et al., 2005; Vaiksaar et al., 2011b; Vaiksaar et al., 2011a; Isacco et al., 2015; Schaumberg et al., 2017; Gordon et al., 2018; Schaumberg et al., 2020; Nakamura and Nose-Ogura, 2021; Gomes et al., 2022) indicated less than 6 months as the duration of COC exposure among participants, whereas 15 studies (Umlauff et al., 2021; Lynch and Nimmo, 1998; Giacomoni and Falgairette, 2000; Lynch et al., 2001; Procter-Gray et al., 2008; Rechichi et al., 2008; Santos et al., 2008; Rebelo et al., 2010; Joyce et al., 2013; Ihalainen et al., 2019; Larsen et al., 2020; Mattu et al., 2020; Bozzini et al., 2021; Taipale-Mikkonen et al., 2021; Notbohm et al., 2023b) indicated more than 6 months as the duration of COC exposure among participants, while only one study did not indicate the duration of COC exposure (Timmons et al., 2005).

### 3.2 Methodological quality of the included studies

All of the included studies were rated as of moderate quality in the overall quality assessment. The average score for RCTs was 8.8/13 with a moderate quality rating, while the cross-sectional studies were also rated moderate with an average score of 6.4/8. The cohort studies were rated high, with an average score of 9.4/11. Briefly, the inter-rater agreement for RCT studies indicated a substantial agreement *k* = 0.61 (CI = 0.37, 0.86), while for cross-sectional studies the inter-rater agreement also indicates a substantial agreement *k* = 0.61 (CI = 0.41, 0.82), whereas the inter-rater agreement for cohort studies indicates a moderate agreement *k* = 0.51 (CI = 0.35, 0.66). Most problems encountered in the quality assessment scores across the RCT studies are attributed to the insufficient number of participants, lack of assessor blinding, and inadequate allocation concealment. Those of the cross-sectional and cohort studies are associated with strategies to address randomization, confounding factors, small sample size, and incomplete follow-up among participants (**Supplementary Table 2**).

### 3.3 Impact of COC on the cardiorespiratory function of premenopausal women engaged in exercise

#### 3.3.1 Heart rate

Overall, 20 studies reported the effect of COC on heart rate (HR) levels in premenopausal women engaged in exercise, as depicted in **Table 1**. A total of 11 studies with an independent comparator group show no effect of COC on the participants’ HR during exercise compared with the non-COC group (Santos et al., 2008; Rebelo et al., 2010; Vaiksaar et al., 2011b; Schaumberg et al., 2017; Gordon et al., 2018; Mattu et al., 2020; Schaumberg et al., 2020 ,Lynch and Nimmo, 1998; Lebrun et al., 2003; Joyce et al., 2013; Isacco et al., 2015), whereas only one cohort study reported a significant increase in HR at submaximal intensities among participants performing exercise and exposed to COC (ethinyl estradiol/progestin) within the dosage range of 15 µg–3 mg compared with non-COC (Taipale-Mikkonen et al., 2021). Furthermore, in the seven studies without an independent comparator group, COC has no effect on the participants’ HR during exercise when compared with the menstrual cycle and the inactive pill phase (Giacomoni and Falgairette, 2000; Lynch et al., 2001; Casazza et al., 2002; Redman et al., 2005; Rechichi et al., 2008; Vaiksaar et al., 2011a; Nakamura and Nose-Ogura, 2021). In contrast, one cross-over study showed that participants on COC (ethinyl estradiol/progestin) within the dosage range of 15 µg–2 mg demonstrated a reduced HR during exercise compared with pre- and post-exercise conditions (Gomes et al., 2022).

**Table 1:** Characteristics of the included studies (n = 29).

Author, year, countryountry	Main finding	Study design	Type of hormonal contraceptive	Dosage	Duration of use	Type of exercise	Exercise intensity	Exercise equipment	Sample size	Participants	Age of participants (years)	Outcome measurement tool
Notbohm et al., 2023, Germany (Notbohm et al., 2023b)	IL-1ra levels are similar between COC vs. non-COC (*p >* 0.05). COC increased IL-1ra levels post-exercise from baseline (+51.1% ± 59.4%, *p* = 0.189), with a concomitant decrease after 24 h post-exercise (–20.5% ± 13.5%, *p* = 0.011) IL-1β levels decreased after 24 h post-exercise when compared with non-COC (*p* = 0.05) IL-8 increased 24 h post-exercise across groups (+66.6% ± 96.3%, *p* = 0.004) but was not significant between groups (*p* = 0.921) IL-6 (*p* = 0.723) and IL-10 (*p* = 0.269) levels remained unchanged across the groups	Cohort study	Monophasic COC	30 μg EE/20 μg CMA, DNG	>9 months	Aerobic/anaerobic	Low-moderate	Smith machine, cycle ergometer, T-FORCE system	COC: 8 Non-COC: 13	Recreationally active women	COC: 22 ± 3 Non-COC: 24 ± 4	ELISA system
Gomes et al., 2022, Brazil (Gomes et al., 2022)	COC reduced resting HR by 16% (*p* < 0.01) and CRP levels by 50% (*p =* 0.04), while VO_2_ max increased by 45% (*p* < 0.01) in the post-exercise period from the baseline and inactive period without changes in the lipid profile (*p* > 0.05)	Cross-sectional	Monophasic COC	20 µg EE/100 µg LNG; CPA 2 mg/EE 0.035 mg; 15–30 µg EE/60–75 µg GSD	6 months	Aerobic	Moderate-high	Treadmill	COC: 12 Non-COC: NR	Recreationally active women	COC: 24 ± 2.5 Non-COC: NR	Polar pulse cardiofrequency meter, enzymatic/turbidimetry method
Bozzini et al., 2021, USA (Bozzini et al., 2021)	COC moderately increase IL-6 (ES: 0.66, *p* = 0.84) vs. non-COC (ES: 0.46, *p* = 0.96) and TNF- α (ES: 0.67, *p* = 0.96) vs. non-COC (ES: 0.07, *p* = 0.63). CRP levels increase substantially (ES: 0.85, *p* = 0.99) vs. non-COC (ES: 0.1, *p* = 0.7). No change in body composition between the groups	Cross-sectional	COC	NR	1 year	Aerobic	Moderate-high	Contact mat, treadmill	COC: 6 Non-COC: 17	Collegiate female soccer players	COC: 19 ± 1 Non-COC: 19 ± 1	COSMED Quark CPET gas analyzer, Polar TeamPro HR monitor, Quest Diagnostics
Taipale-Mikkonen et al., 2021, Finland (Taipale-Mikkonen et al., 2021)	HR at the aerobic threshold tended to be lower in the non-COC vs. the COC group (*p* = 0.026). No phase-related differences in VO_2_peak (*p* = 0.889) and blood lactate (*p* = 0.312) were observed at the aerobic threshold in either group	Cohort study	Monophasic COC	0.02 mg EE/3 mg DRSP; 0.03 mg EE/3 mg DRSP; NOMAC 2.5 mg/E2 1.5 mg; CPA 2 mg/EE 0.035 mg; ENG 0.120 mg/EE 0.015 mg	1 year	Aerobic/anaerobic	Moderate-high	Treadmill	COC: 11 Non-COC: 17	Recreationally active women	COC: 23 ± 2 Non-COC: 26 ± 4	Polar 800 HR monitor, Oxycon mobile gas analyzer, EKF diagnostic, C-line system, InBody 720 analyzer
Umlauff et al., 2021, Germany (Umlauff et al., 2021)	BLa levels were higher in the COC vs. non-COC groups (*p* = 0.001). BMI remain unaltered between the groups (*p* = 0.42)	Cohort study	Monophasic COC	30 μg EE–20 μg CMA/DNG	>9 months	Anaerobic/aerobic	Moderate-high	Smith machine, cycle ergometer, T-FORCE system	COC:8 Non-COC: 13	Recreationally active women	COC: 22 ± 3 Non-COC: 24 ± 4	Biosen S-line analyzer, smartLAB fit1 digital scale and Akern Srl body analyzer
Larsen et al., 2020, Australia (Larsen et al., 2020)	COC increased CRP levels significantly (*p* < 0.001) vs. non-COC. The levels of other cytokines and PBMC immune cell subsets were similar between the groups, except for a slight decrease in IL-6 (*p* = 0.062). BMI was also similar between the groups	Cross-sectional	Monophasic COC	NR	>1 year	Anaerobic/aerobic	Moderate-high	NR	COC: 25 Non-COC: 28	Elite female Olympic athletes	OC: 24.7 ± 3.5 Non-COC: 24.4 ± 4	Immunoturbidimetric assay/COBAS Integra 400 system, Bioplex 200 suspension array reader, CyTOF 2 Helios
Mattu et al., 2020, Canada (Mattu et al., 2020)	During maximal exercise, no detectable difference in the physiological variables (HR, V̇O_2_, V̇CO_2_, V̇E, and BLa {30 min}) between the groups and across the phases were observed (*p* > 0.05). RER decreases significantly across the phases in the groups (p = 0.035), but not between the groups (*p* = 0.49)	Cross-sectional	Monophasic COC	20–35 µg EE/100–200 µg LNG/DSG	>6 months	Aerobic/anaerobic	Moderate-high	Cycle ergometer	COC: 15 Non-COC: 15	Recreationally active and highly trained women	OC: 24 ± 4 Non-COC: 27 ± 6	Quark CPET, COSMED, Garmin (chest strap) HR monitor, EKF Biosen C-Line analyzer
Nakamura and Nose-Ogura, 2020, Japan (Nakamura and Nose-Ogura, 2021)	Short-term COC use and the menstrual cycle phase did not affect body composition, HR, or VO_2_ in female athletes (*p* > 0.05). There is a phase effect of the menstrual cycle (*p* < 0.05) on the BLa levels during anaerobic capacity (Wingate test), unlike aerobic capacity. No change in BLa level between the COC and the menstrual phase of the female athletes (*p* > 0.05)	Cross-sectional	Monophasic COC	20 μg EE/1 mg NET	<6 months	Aerobic/anaerobic	Moderate-high	Cycle ergometer	COC:10 Non-COC: NR	Female athletes	COC: 23 ± 4.1 Non-COC: NR	Polar Electro RS-800CX HR monitor, AE-310S gas analyzer, Lactate Pro II test meter/Biosen S-Line analyzer
Schaumberg et al., 2020, Australia (Schaumberg et al., 2020)	Improved HR responses after training are similar between the COC and non-COC groups at moderate and high exercise intensities across time points (*p* > 0.05). Similarly, there is no difference in oxygen uptake between the COC and non-COC groups at any time point or exercise intensity (*p* > 0.05). However, non-COC demonstrated improved oxygen uptake post-training from baseline during moderate (*p* = 0.021) and high (*p* = 0.015) exercise intensities	Cross-sectional	Monophasic COC	20–30 µg EE/100–150 µg LNG; 20–30 µg EE/75 µg GSD; 20 µg EE/150 µg DSG	6 months	Aerobic/anaerobic	Moderate-high	Cycle ergometer	COC:25 Non-COC: 22	Recreationally active women	COC: 25.5 ± 5.4 Non-COC: 26.4 ± 5.2	TrueOne 2400 Indirect Calorimetry System, PhysioFlow
Ihalainen et al., 2019, Finland (Ihalainen et al., 2019)	The changes in hs-CRP levels across the time points are significantly greater in the COC vs. the non-COC groups (*p* = 0.015). Lipid profile, IL-6, and TNF-α remained unaltered in both groups (*p* > 0.05)	Cross-sectional	Monophasic COC	NR	1 year	Aerobic/anaerobic	Low-high	NR	COC: 9 Non-COC: 9	Recreationally active women	COC: NR Non-COC: NR	Sysmex KX-21N, spectrophotometry, Immulite 1000
Gordon et al., 2017, UK (Gordon et al., 2018)	There were no significant differences in VO_2_ max or associated cardiodynamic responses between COC and non-COC groups across the phases (*p* > 0.05). However, ΔVO_2_ in the final 60s of VO_2_ max testing declines to 0% in the COC groups compared to the non-COC group (55%) in the luteal phase (*p* < 0.05)	Cohort study	Monophasic COC	30 μg EE/150 μg LNG	<6 months	Aerobic/anaerobic	Moderate-high	Cycle ergometer	COC: 6 Non-COC: 10	Recreationally active women	COC: 20.6 ± 1.6 Non-COC: 21.7 ± 2.2	Cortex 3B Metalyzer, Physioflow, Opti CCA-TS, GM7 Micro-Stat analyzer
Schaumberg et al., 2017, Australia (Schaumberg et al., 2017)	Improved VO_2_ peak after SIT is higher in the non-COC (+13.0%) vs. the COC group (+8.5%) (*p* = 0.010). However, in the 4-week follow-up, the decline in VO_2_ peak adaptation was spared in the COC (─ 4.0%) vs. the non-COC group (─ 7.7%) (*p* = 0.010)	Cross-sectional	Monophasic COC	20–30 µg EE/100–150 µg LNG; 20–30 µg EE/75 µg GSD; 20 µg EE/150 µg DSG	6 months	Anaerobic/aerobic	Moderate- high	Cycle ergometer	COC:25 Non-COC: 16	Recreationally active women	COC: 25.5 ± 5.4 Non-COC: 27.6 ± 5.4	TrueOne 2400 Indirect Calorimetry System, PhysioFlow
Isacco et al., 2015, France (Isacco et al., 2015)	Cardiorespiratory parameters at the AT and at the maximal aerobic exercise capacity are similar between the COC and non-COC groups (*p* > 0.05). COC increased lipid profile (TC, LDL-c, and TG) when compared with non-COC (*p* < 0.05)	Cohort study	Monophasic COC	20 μg EE/75 μg GSD; 30 μg EE/150 μg LNG	6 months	Aerobic/anaerobic	Moderate-high	Cycle ergometer	COC: 11 Non-COC: 10	Recreationally active women	COC: 21.2 ± 1.9 Non-COC: 22.9 ± 3.6	Oxycon pro-Delta gas analyzers, Konelab 20 analyzer
Joyce et al., 2013, Australia (Joyce et al., 2013)	Long-term COC use slightly decreases peak VO_2_ (2.13 ± 0.2 L. min^─^ 1) and submaximal VO_2_ (1.18 ± 0.15 L. min^─^ 1) at the AT compared with non-COC (2.59 ± 0.5 L. min^─^ 1; 1.47 ± 0.27 L. min^─^ 1; *p* < 0.05). However, HR, VE, and BLa remained constant across groups (*p* > 0.05)	Cohort study	Monophasic COC	20 µg EE/150 µg LNG; CPA 2 mg/EE 0.035 mg	>1 year	Aerobic/anaerobic	Moderate-high	Cycle ergometer	COC: 8 Non-COC: 8	Recreationally active women	COC: 20 ± 2 Non-COC: 22 ± 3	MedGraphics Ultima CardiO_2_ gas analyzer, X12 + 5-lead electrode configuration
Vaiksaar et al., 2011, Estonia (Vaiksaar et al., 2011a)	COC cycle phases do not significantly impact VE (ES: 0.1; *p* = 0.82), HR (ES: 0.1; *p* = 0.73), VO_2_ (ES: 0.0; *p* = 0.74), and BLa (ES: 0.3; *p* = 0.31) during the 1-h rowing ergometer exercise among recreationally trained female rowers	Cohort study	Monophasic COC	20 μg EE/75 μg GSD	<6 months	Aerobic	Moderate-high	Rowing ergometer	COC: 8 Non-COC: NR	Recreationally trained female rowers	COC: 21.0 ± 2.8 Non-COC: NR	Cortex MetaMax I open-circuit spirometry system, Sporttester Polar 725X, and Lange enzymatic measurement system
Vaiksaar et al., 2011, Estonia (Vaiksaar et al., 2011b)	COC and menstrual cycle phases do not affect VO_2_ or HR during maximal aerobic or during the aerobic–anaerobic transition (*p* > 0.05)	Cohort study	Monophasic COC	20 μg EE/75 μg GSD	<6 months	Aerobic/anaerobic	Moderate-high	Rowing ergometer	COC: 9 Non-COC: 15	Recreationally trained and competitive female rower athletes	COC: 21.0 ± 2.6 Non-COC: 18.4 ± 2	Cortex MetaMax I open-circuit spirometry system, Sporttester Polar 725X, and Lange enzymatic measurement system
Rebelo et al., 2010, Brazil (Rebelo et al., 2010)	Long-term use of COC does not alter the V̇O_2_, V̇CO_2_, VE, HR, and RER at the peak and the AT level of exercise when compared with non-COC (*p* > 0.05)	Cross-sectional	Monophasic COC	20 μg EE/75 μg GSD	>1 year	Aerobic	Moderate-high	Cycle ergometer	COC: 23 Non-COC: 20	Recreationally active university Students	COC: 23.1 ± 2.9 Non-COC: 20.5 ± 3.8	Instramed MINISCOPE II HR monitor, CPXD metabolic analyzer
Procter-gray et al., 2008, USA (Procter-Gray et al., 2008)	COC use does not cause weight or fat mass gain among young female runners when compared with non-COC	RCT	Monophasic COC	30 μg EE/0.3 mg NORG	>6 months	Aerobic/anaerobic	Moderate-high	NR	COC: 69 Non-COC: 81	Competitive female runners	OC: 22.3 ± 2.7 Non-COC: 21.9 ± 2.6	Standard stadiometers and balance beam scales
Rechichi et al., 2008, Australia (Rechichi et al., 2008)	COC does not affect HR, VO_2_ peak, blood glucose, or RER across the pill phases of the endurance test (1-h cycle) (*p* > 0.05). However, VE and BLa were significantly higher during COC than during the inactive phases (*p* < 0.05)	Cohort study	Monophasic COC	20–30 µg EE/100–150 µg LNG; 30 µg EE/150 µg DSG; 0.03 mg EE/3 mg DRSP; CPA 2 mg/EE 0.035 mg; 30 µg EE/500 µg–1 mg NET	6–120 months	Aerobic	Moderate-high	Cycle ergometer	COC: 13 Non-COC: NR	Female cyclists or triathletes	COC: 34 ± 7 Non-COC: NR	Ametek gas Analyzers, Morgna turbine ventilometer, Polar Vantage HR monitor, ABLTM 700 Series radiometer
Santos et al., 2008, Brazil (Santos et al., 2008)	The HR, VO_2_, VCO_2_, and VE were similar between COC and non-COC during maximal and AT exercise (*p* > 0.05). COC increased lipid profile (TG and TC) when compared with non-COC (*p* < 0.05)	Cross-sectional	Monophasic COC	20 µg EE/75 µg GSD; CPA 2 mg/EE 0.035 mg; 0.03 mg EE/3 mg DRSP	>6 months	Aerobic/anaerobic	Moderate-high	Cycle ergometer	COC: 10 Non-COC: 10	Recreationally active women	COC: NR Non-COC: NR	Instramed MINISCOPE II HR monitor, CPXD metabolic analyzer, automatic chloromimetric/enzymatic method
Redman et al., 2005, Australia (Redman et al., 2005)	COC did not alter HR, VO_2_, VE, VCO_2_, or BLa levels during submaximal exercise. However, high COC decreased RER levels(*p* < 0.05) compared to low COC. High COC increased VO_2_ compared with low COC (*p* < 0.05), without altering HR, VE, VCO_2_, RER, and BLa levels during incremental exercise	RCT	Monophasic COC	35 μg EE/0.5–1 mg NET	6 months	Aerobic/anaerobic	Moderate-high	Cycle ergometer	COC: 23 Non-COC: NR	Recreationally active women	COC: 22.6 ± 3.5 Non-COC: NR	Electrocardiograph, ventilometer, and gas analyzers, ABL System 620 radiometer
Timmons et al., 2005, Canada (Timmons et al., 2005)	The changes in immune cell counts, particularly neutrophils, were significantly greater in COC than in non-COC, regardless of MP phase (*p* < 0.05). The change in IL-6 level is comparable between COC and non-COC across the menstrual phases (*p* > 0.05)	Cohort study	Triphasic COC	35 μg EE/NGM 180–215–250 μg; 30–40 μg EE/LNG 50–75–125	NR	Aerobic	Moderate-high	Cycle ergometer	COC: 6 Non-COC: 6	Recreationally active women	COC: 22 ± 3 Non-COC: 21 ± 2	Moxus Modulator open-circuit gas analyzer, ELISA system, automated Coulter counter
Lebrun et al., 2003, Canada (Lebrun et al., 2003)	COC decreased VO_2_ max (relative: *p* = 0.019 and absolute: *p* = 0.05) by 4.7% compared with non-COC (placebo), with a 1.5% decrease across the menstrual phases. Other variables, such as HR, VE, RER, and hematocrit levels, remained constant (*p* > 0.05)	RCT	Triphasic COC	35 μg EE/0.5–1 mg NET	2 months	Aerobic/anaerobic	Moderate-high	Treadmill	COC: 7 Non-COC: 7	Female athletes	COC: NR Non-COC: NR	Polar Vantage HR monitor, Beckman metabolic measurement cart
Casazza et al., 2002, USA (Casazza et al., 2002)	After 4 months, COC decreased absolute (11%) and relative (13%) VO_2_ peak and VCO_2_ peak production (15%) from baseline in the inactive pill phase (*p* < 0.05). Other variables, such as HR, VE, and RER, remained constant	Cohort study	Triphasic COC	0.035 mg EE/0.18 NORG; 0.035 mg EE/0.215 NORG; 0.035 mg EE/0.25 NORG	4 months	Aerobic/anaerobic	Moderate-high	Cycle ergometer	COC: 6 Non-COC: NR	Recreationally active women	COC: 25.5 ± 1.5 Non-COC: NR	Ametek open-circuit gas analyzer, Quinton Q750 electrocardiograph
Lynch et al., 2001, Scotland (Lynch et al., 2001)	Low-dose COC did not alter the HR, VO_2_ max, and other metabolic variables such as blood glucose, BLa, and FFA across the pill phase cycle (*p* > 0.05)	Cohort study	Monophasic COC	30 μg EE/150 μg LNG; 20 μg EE/150 μg LNG; 35 μg EE/500 μg NET	1 year	Aerobic	Moderate	Treadmill	COC: 9 Non-COC: NR	Recreationally active women	COC: 23.1 ± 4 Non-COC: NR	Polar Electro-3000 HR monitor, QP9000 open-circuit mass-spectrometer, fluori-metric assay
Giacomoni and Falgairette, 2000, UK (Giacomoni and Falgairette, 2000)	COC did not alter HR, VE, or VCO_2_ across the pill phase (*p* > 0.05). VO_2_ levels decreased in the early (3%) and late (5%) phases of COC use (*p* = 0.008), while RER increased in the early phase of COC use compared to the inactive pill phase (*p* < 0.05), regardless of the exercise stage	RCT	Monophasic COC	30 μg EE/75 μg GSD; 20 μg EE/150 μg DSG	18 months	Aerobic	Moderate	Treadmill	COC: 10 Non-COC: NR	Recreationally active women	COC: 23 ± 3 Non-COC: NR	Sensor Medics 2900 open-circuit respiratory system, Polar Electro-4000 HR monitor
Lynch and Nimmo, 1998, Scotland (Lynch and Nimmo, 1998)	HR, VO_2_ max, and Bla levels are similar between COC and non-COC group (*p* > 0.05)	Cohort-study	Monophasic COC	30–35 μg EE/150 μg LNG; 250 μg NGM	>1 year	Aerobic/anaerobic	Moderate-high	Treadmill	COC: 5 Non-COC: 10	Recreationally active women	COC: 22.2 ± 0.4 Non-COC: 26.8 ± 7	Polar Electro-3000 HR monitor, online Oxycongamma gas analyzer, fluorimetric assay
Bryner et al., 1996, USA (Bryner, 1996)	VO_2_ max and breathing frequency during a maximal treadmill test or endurance run are similar in both low-dose COC and non-COC regardless of menstrual cycle or pill phase (*p* > 0.05)	Cohort study	Monophasic COC	35 μg EE/1 mg NET	<6 months	Aerobic/anaerobic	Moderate-high	Treadmill	COC: 7 Non-COC: 3	Recreationally active women	COC: NR Non-COC: NR	Sensormedics OM-II and LB-2 analyzers, electrocardiograph
Notelovitz et al., 1986, USA (Notelovitz et al., 1987)	Low-dose COC decreased VO_2_ max significantly by 8% compared with non-COC, while the lipid profile remained unaltered (*p* > 0.05)	Cross-sectional	Monophasic COC	35 μg EE/400 μg NET	6 months	Aerobic	Moderate-high	Treadmill	COC: 6 Non-COC: 6	Recreationally active women	COC: 24.5 ± 3.2 Non-COC: 24.3 ± 3	Palpitation, Beckman metabolic measurements cart

NR, not reported; COC, combined oral contraceptives; EE/E2, ethinylestradiol; DSG, desogestrel; DNG, dienogest; DRSP, drospirenone; ENG, etonogestrel; CMA, chlormadinone acetate; LNG, levonorgestrel; MPA, medroxyprogesterone acetate; GSD, gestodene; RCT, randomized control trial; NOMAC, nomegestrol acetate; CPA, cyproterone acetate; VO_2_, oxygen consumption; VCO_2_, carbon dioxide production; NORG, norgestrel; VE, minute ventilation; NGM, norgestimate; IL, interleukin; PBMC, peripheral blood mononuclear cell; CRP, C-reactive protein; NET, norethisterone; TNF-α, tumor necrosis factor-alpha; ES, effect size; USA, United States of America; UK, United Kingdom; BLa, blood lactate; SIT, sprint interval training; AT, anaerobic threshold; ELISA, enzyme-linked immunosorbent assay; CMJ, counter-movement jump; CPET, cardiopulmonary exercise test; TG, triglyceride; TC, total cholesterol; LDL-c, low-density lipoprotein-cholesterol.

First generation: NET, CMA, CPA, and MPA; second generation: LNG, NORG, and ENG; third generation: GSD, DSG, and NGM; and fourth generation: DRSP, DNG, and NOMAC.

**Figure 3:**
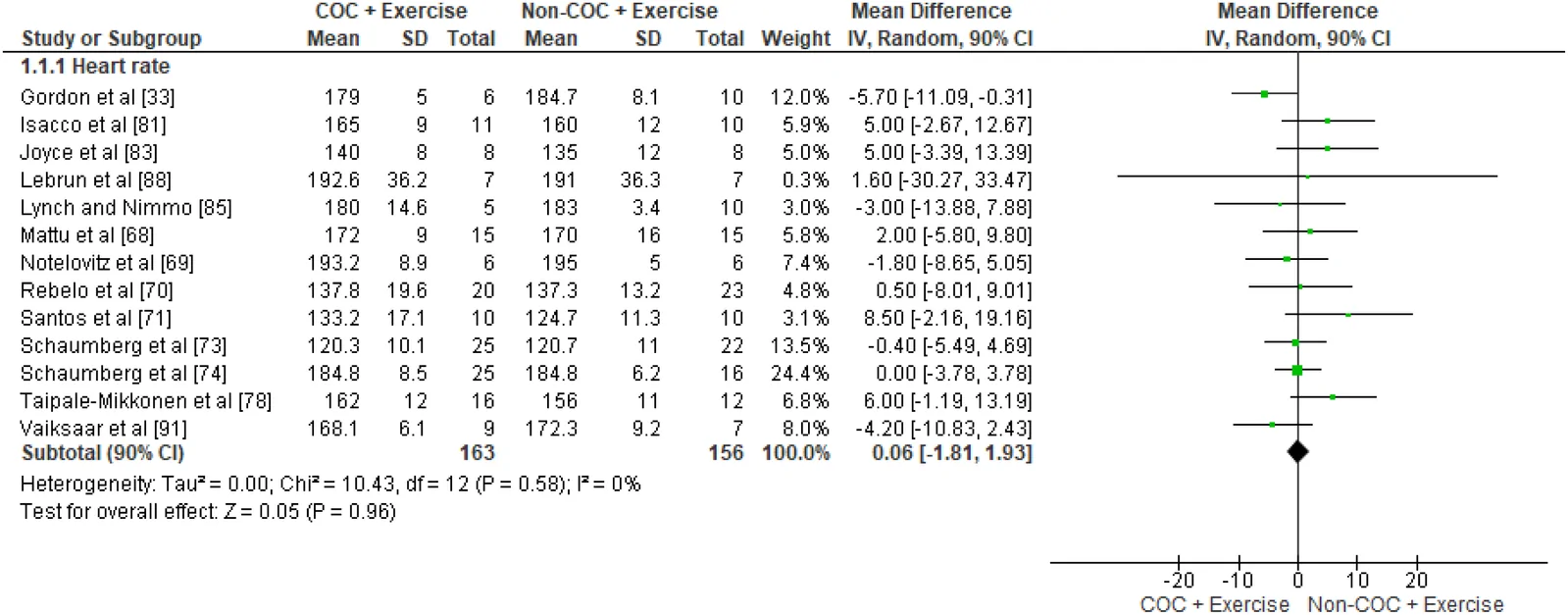
Forest plot showing the impact of COC on the heart rate of premenopausal women involved in exercise when compared with non-COC.

Nonetheless, the overall pooled estimate of the meta-analysis involving studies with an independent comparator group showed little to no difference in the effect of COC on the participants’ heart rate when compared to the non-COC group with a small effect size [MD = 0.06, 95% CI (−1.81, 1.93), *p* = 0.96, a low level of heterogeneity (*I*^2^ = 0%, *p* = 0.58)] (low certainty evidence) (**Figure 3**). Thus, the result showed that COC use does not alter the HR level in premenopausal women engaged in exercise.

#### 3.3.2 Submaximal oxygen consumption

Overall, 11 studies reported the effect of COC on submaximal oxygen consumption (VO_2_) levels in premenopausal women engaged in exercise, as depicted in **Table 1**. Seven studies with an independent comparator group showed that the effect of COC (ethinyl estradiol/progestin) within the dosage range of 15 µg–3 mg on VO_2_ in premenopausal women engaged in exercise is comparable to that of the non-COC group (Lebrun et al., 2003; Rebelo et al., 2010; Vaiksaar et al., 2011b; Isacco et al., 2015; Mattu et al., 2020; Schaumberg et al., 2020; Taipale-Mikkonen et al., 2021). In contrast, only one cohort study showed that the long-term use of COC (ethinyl estradiol/progestin) at a dosage range of 15 µg–2.5 mg reduces the VO_2_ levels in premenopausal women engaged in exercise compared with the non-COC group (Joyce et al., 2013). In the two studies without an independent comparator group, both showed that COC reduced the VO_2_ levels during exercise in premenopausal women when compared with the inactive pill phase (Giacomoni and Falgairette, 2000; Casazza et al., 2002). Evidence from a longitudinal cohort study suggests a 13% reduction in VO_2_ after 4 months of COC use compared with baseline and the inactive pill phase (Casazza et al., 2002). Similarly, evidence from a randomized crossover trial (RCT) showed a decrease in VO_2_ levels in the early (3%) and late (5%) phases of COC use in participants compared with the inactive pill phase during the exercise (Giacomoni and Falgairette, 2000). In contrast, only one cohort study reported no changes in VO_2_ levels across the pill cycles among participants exposed to COC who engaged in exercise (Vaiksaar et al., 2011a).

**Figure 4:**
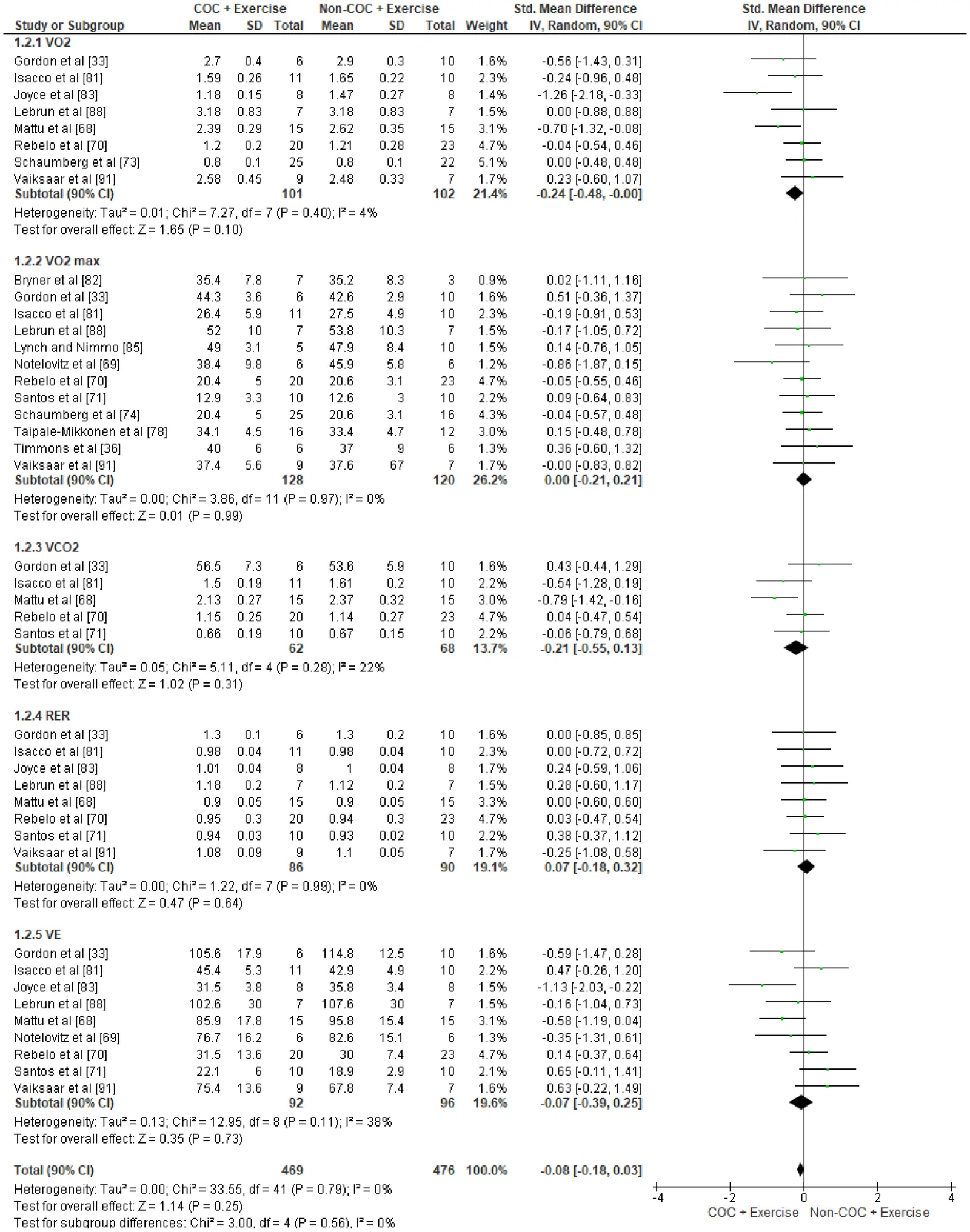
Forest plot showing the impact of COC on respiratory changes in premenopausal women engaged in exercise compared with non-COC users. VO_2_, submaximal oxygen consumption; VO_2_ max, maximal oxygen consumption; VCO_2_, carbon dioxide production; RER, respiratory exchange ratio; VE, minute ventilation.

In the overall pooled estimate of the meta-analysis of studies with an independent comparator group, there was little to no difference in VO_2_ level in those taking COC when compared to the non-COC group with a small effective size [SMD = -0.24, 95% CI (−0.48, 0.00), *p* = 0.10, a low level of heterogeneity (*I*^2^ = 4%, *p* = 0.40)] (low certainty evidence) (**Figure 4**). Thus, the result showed that COC use does not alter the VO_2_ level in premenopausal women engaged in exercise.

#### 3.3.3 Maximal oxygen consumption

Overall, 20 studies reported the effect of COC on maximal oxygen consumption (VO_2_ max) in premenopausal women engaged in exercise, as shown in **Table 1**. Nine studies with an independent comparator group reported comparable VO_2_ max levels in premenopausal women engaged in exercise and exposed to COC (ethinyl estradiol/progestin) within the dosage range of 15 µg–3 mg compared with the non-COC group (Bryner, 1996; Lynch and Nimmo, 1998; Santos et al., 2008; Rebelo et al., 2010; Vaiksaar et al., 2011b; Isacco et al., 2015; Schaumberg et al., 2017; Gordon et al., 2018; Taipale-Mikkonen et al., 2021). In contrast, four studies showed a reduction in VO_2_ max among premenopausal women who exercised and used COC (ethinylestradiol/progestin) at doses of 20 µg–2.5 mg compared with the non-COC group (Notelovitz et al., 1987; Lebrun et al., 2003; Joyce et al., 2013; Schaumberg et al., 2020). In studies without an independent comparator group, four studies reported comparable differences in VO_2_ max levels between the COC pill cycle and inactive pill phase among premenopausal women engaged in exercise (Lynch et al., 2001; Rechichi et al., 2008; Vaiksaar et al., 2011a; Nakamura and Nose-Ogura, 2021). In contrast, evidence from a longitudinal cohort study showed that triphasic COC decreased the VO_2_ max levels by 11% after 4 months from the baseline and inactive pill phase (Casazza et al., 2002). Contrastingly, two studies showed increased VO_2_ max in premenopausal women who engaged in exercise and were exposed to a monophasic COC (ethinylestradiol/progestin) within the dosage range of 15 µg–2 mg (Redman et al., 2005; Gomes et al., 2022). Evidence from a cross-sectional study suggests a 45% increase in VO_2_ max in the post-exercise period relative to baseline and the inactive period in participants on COC (Gomes et al., 2022). Similarly, evidence from a single-blind, randomized, counterbalanced, crossover study showed that a high-dose progestin COC increased VO_2_ max during incremental exercise compared with a low-dose progestin COC in premenopausal women (Redman et al., 2005). In this study, the ethinylestradiol dose in the COC was constant, whereas the progestin dose varied twofold, indicating a dose-dependent effect of the COC on VO_2_ max (Redman et al., 2005).

In the overall pooled estimate of the meta-analysis, studies with an independent comparator group showed little to no difference in VO_2_ max levels in those taking COC when compared to non-COC with a small effect size [SMD = 0.00, 95% CI (−0.21, 0.21), *p* = 0.99, a low level of heterogeneity (*I*^2^ = 0%, *p* = 0.97)] (low certainty evidence) (**Figure 4**). Thus, the result showed that COC use does not alter the VO_2_ max level in premenopausal women engaged in exercise.

#### 3.3.4 Carbon dioxide production

Overall, eight studies reported the effect of COC on carbon dioxide production (VCO_2_) levels in premenopausal women engaged in exercise, as shown in **Table 1**. Five studies with an independent comparator group showed that the impact of COC (ethinyl estradiol/progestin) within the dosage range of 20 µg–3 mg on VCO_2_ levels among premenopausal women engaged in exercise is comparable with that of the non-COC group (Santos et al., 2008; Rebelo et al., 2010; Isacco et al., 2015; Gordon et al., 2018; Mattu et al., 2020). Similarly, two studies without an independent comparator group showed that COC does not alter the VCO_2_ levels in premenopausal women during exercise (Giacomoni and Falgairette, 2000; Redman et al., 2005). Evidence from an RCT study showed that COC does not alter the VCO_2_ levels across the pill phase (Giacomoni and Falgairette, 2000). Similarly, evidence from a single-blind, randomized, counterbalanced, crossover study demonstrates a comparable difference in VCO_2_ levels between COC with a high progestin dose and a low progestin dose in the participants (Redman et al., 2005). In contrast, evidence from a longitudinal cohort study showed that triphasic COC (ethinylestradiol/progestin) within the dosage range of 35 µg–2.5 mg reduced the VCO_2_ levels by 15% in premenopausal women engaged in exercise after 4 months relative to baseline (Casazza et al., 2002).

In the overall pool estimates of the meta-analysis, studies with an independent comparator group showed little to no difference in VCO_2_ levels in those taking COC when compared to non-COC with a small effect size [SMD = -0.21, 95% CI (−0.55, 0.13), *p* = 0.31, a low level of heterogeneity (*I*^2^ = 22%, *p* = 0.28)] (low certainty evidence) (**Figure 4**). Thus, our result showed that COC use does not alter the VCO_2_ level in premenopausal women engaged in exercise.

#### 3.3.5 Respiratory exchange ratio

Overall, 13 studies reported the effect of COC on respiratory exchange ratio (RER) levels in premenopausal women engaged in exercise, as shown in **Table 1**. Eight studies with an independent comparator group showed that the impact of COC (ethinyl estradiol/progestin) use within the dosage range of 20 µg–3 mg on RER among premenopausal women engaged in exercise was comparable to that of the non-COC group (Lebrun et al., 2003; Santos et al., 2008; Rebelo et al., 2010; Vaiksaar et al., 2011b; Joyce et al., 2013; Isacco et al., 2015; Gordon et al., 2018; Mattu et al., 2020). Similarly, in the studies without an independent comparator group, three studies showed that COC does not alter the RER across the pill cycle relative to the inactive pill phase in premenopausal women engaged in exercise (Casazza et al., 2002; Rechichi et al., 2008; Vaiksaar et al., 2011a). In contrast, evidence from an RCT study reported a significant increase in RER during early exposure to COC when compared with the inactive pill phase in premenopausal women engaged in exercise (Giacomoni and Falgairette, 2000). However, evidence from a single-blind, randomized, counterbalanced, crossover study found that a high dose of progestin COC decreased the RER levels compared with a low dose during submaximal exercise in premenopausal women (Redman et al., 2005).

In the overall pool estimates of the meta-analysis, studies with an independent comparator group showed that the effect of COC on RER showed little to no difference in those taking COC when compared to the non-COC group with a small effect size [SMD = 0.07, 95% CI (−0.18, 0.32), *p* = 0.64, a low level of heterogeneity (*I*^2^ = 0%, *p* = 0.99)] (low certainty evidence) (**Figure 4**). Thus, the result showed that COC use does not alter the RER level in premenopausal women engaged in exercise.

#### 3.3.6 Minute ventilation

Overall, 16 studies reported the effect of COC on minute ventilation (VE) levels in premenopausal women engaged in exercise, as shown in **Table 1**. A total of 10 studies with an independent comparator group showed that the impact of COC (ethinylestradiol/progestin) use within the dosage range of 20 µg–3 mg on VE levels in premenopausal women engaged in exercise is comparable to that of the non-COC group (Notelovitz et al., 1987; Lebrun et al., 2003; Santos et al., 2008; Rebelo et al., 2010; Vaiksaar et al., 2011b; Joyce et al., 2013; Isacco et al., 2015; Schaumberg et al., 2017; Gordon et al., 2018; Mattu et al., 2020). Similarly, in the studies without an independent comparator group, five studies showed that the impact of COC on VE level is comparable across the pill cycle relative to the inactive pill phase in premenopausal women engaged in exercise (Giacomoni and Falgairette, 2000; Casazza et al., 2002; Redman et al., 2005; Vaiksaar et al., 2011a; Nakamura and Nose-Ogura, 2021). In contrast, evidence from a cohort study showed that COC (ethinylestradiol/progestin) within the dosage range of 20 µg–3 mg increased the VE levels compared with the inactive pill phase in premenopausal women engaged in exercise (Rechichi et al., 2008).

In the overall pool estimates of the meta-analysis of studies with an independent comparator group, there was little to no difference in VE level in those taking COC when compared to the non-COC group with a small effect size [SMD = -0.07, 95% CI (−0.39, 0.25), *p* = 0.73, a low level of heterogeneity (*I*^2^ = 38%, *p* = 0.11)] (low certainty evidence) (**Figure 4**). Thus, the result showed that COC use does not alter the VE level in premenopausal women engaged in exercise.

### 3.4 Impact of COC on the markers of immune response among premenopausal women engaged in exercise

Overall, seven studies reported the effects of COC on immune response markers in premenopausal women who engaged in exercise, as shown in **Table 1**. In the studies with an independent comparator, a cohort study showed that triphasic COC (ethinyl estradiol/progestin) within the dosage range of 20–250 µg increased the immune cell counts in premenopausal women engaged in exercise compared with the non-COC group (Timmons et al., 2005). The between-group exercise response shows that COC users demonstrated significantly greater increases in total leukocyte, neutrophil, monocyte, and lymphocyte counts during the luteal phase compared with the non-COC group. Among these responses, neutrophils showed the most pronounced between-group effect, with exercise-induced increases in neutrophil counts consistently higher in COC users across menstrual phases, although the strongest statistical effect was observed during the luteal phase.

Regarding the acute-phase systemic inflammatory reactants, three studies reported increased levels of CRP in premenopausal women engaged in exercise and exposed to COC (ethinyl estradiol/progestin) at doses of 15 µg–2 mg compared with the non-COC group (Ihalainen et al., 2019; Larsen et al., 2020; Bozzini et al., 2021). Similarly, evidence from a cross-sectional study without an independent comparator showed increased CRP levels in the post-exercise period relative to baseline and the inactive period in premenopausal women on COC (Gomes et al., 2022).

In the cytokine milieu, two studies with an independent comparator showed that the IL-1ra levels were similar in premenopausal women who exercised and were exposed to COC compared with the non-COC group (Larsen et al., 2020; Notbohm et al., 2023b). During the pill cycle phase of the cohort study, COC significantly decreased the IL-1ra and IL-1β levels 24 h after exercise in participants (Notbohm et al., 2023b). However, the IL-8 levels increased significantly at 24 h post-exercise across groups, although not significantly between groups (Notbohm et al., 2023b). Similarly, four studies showed that the IL-6, IL-10, and TNF-alpha levels were similar in premenopausal women who exercised and were exposed to COC compared with the non-COC group (Timmons et al., 2005; Ihalainen et al., 2019; Larsen et al., 2020; Notbohm et al., 2023b). In contrast, a cross-sectional study showed a moderate increase in IL-6 and TNF-α levels among premenopausal women who exercised and were exposed to COC compared with the non-COC group (Bozzini et al., 2021). Moreover, evidence from a cohort study showed that COC decreased the IL-1β levels 24 h post-exercise in premenopausal women who were exposed to COC compared with the non-COC group (Notbohm et al., 2023b).

**Figure 5:**
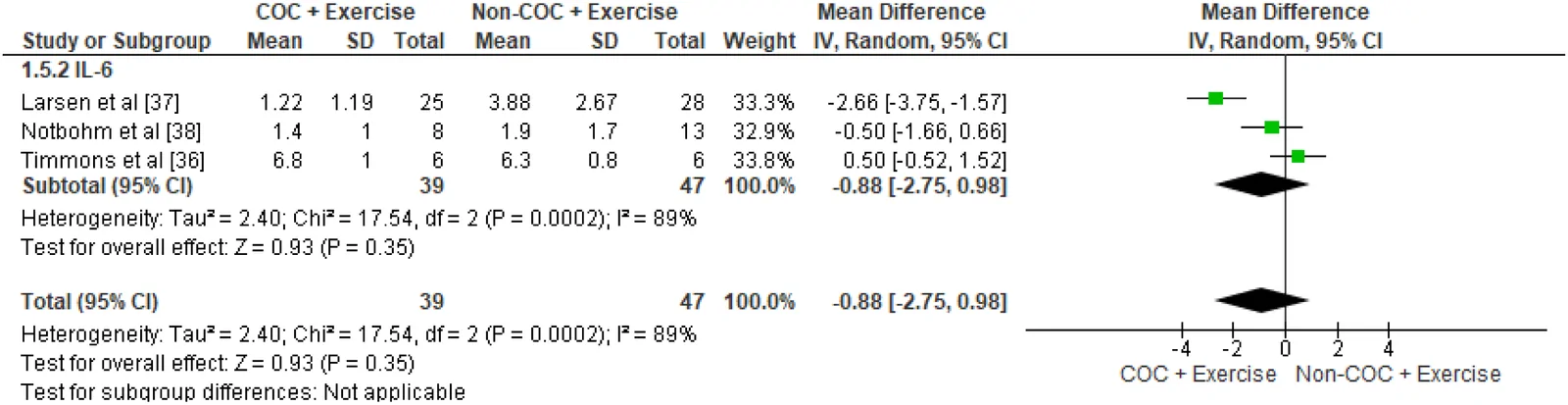
Forest plot showing the impact of COC on interleukin-6 levels in premenopausal women engaged in exercise compared with non-COC users.

In the overall pool estimates of the meta-analysis of studies with an independent comparator, there was little to no difference in IL-6 levels in those taking COC when compared to the non-COC group with a large effect size [SMD = −0.88, 95% CI (−2.75, 0.98), *p* = 0.35 and a high level of heterogeneity (*I*^2^ = 89%, *p* = 0.0002)] (low certainty evidence) (**Figure 5**). Thus, the result showed that COC use does not alter the IL-6 levels in premenopausal women engaged in exercise.

### 3.5 Impact of COC on the markers of body composition and metabolic changes among premenopausal women engaged in exercise

#### 3.5.1 Body mass index

Overall, 11 studies reported the effect of COC on body mass index (BMI) in premenopausal women engaged in exercise, as shown in **Table 1**. Nine studies with an independent comparator showed a comparable BMI in premenopausal women engaged in exercise and exposed to COC (ethinylestradiol/progestin) within the dosage range of 15 µg–2 mg compared with the non-COC group (Procter-Gray et al., 2008; Rebelo et al., 2010; Vaiksaar et al., 2011b; Joyce et al., 2013; Isacco et al., 2015; Schaumberg et al., 2017; Larsen et al., 2020; Schaumberg et al., 2020; Notbohm et al., 2023b). Moreover, two studies without an independent comparator showed unaltered BMI in premenopausal women engaged in exercise throughout the COC pill cycle and inactive pill phase (Redman et al., 2005; Gomes et al., 2022).

**Figure 6:**
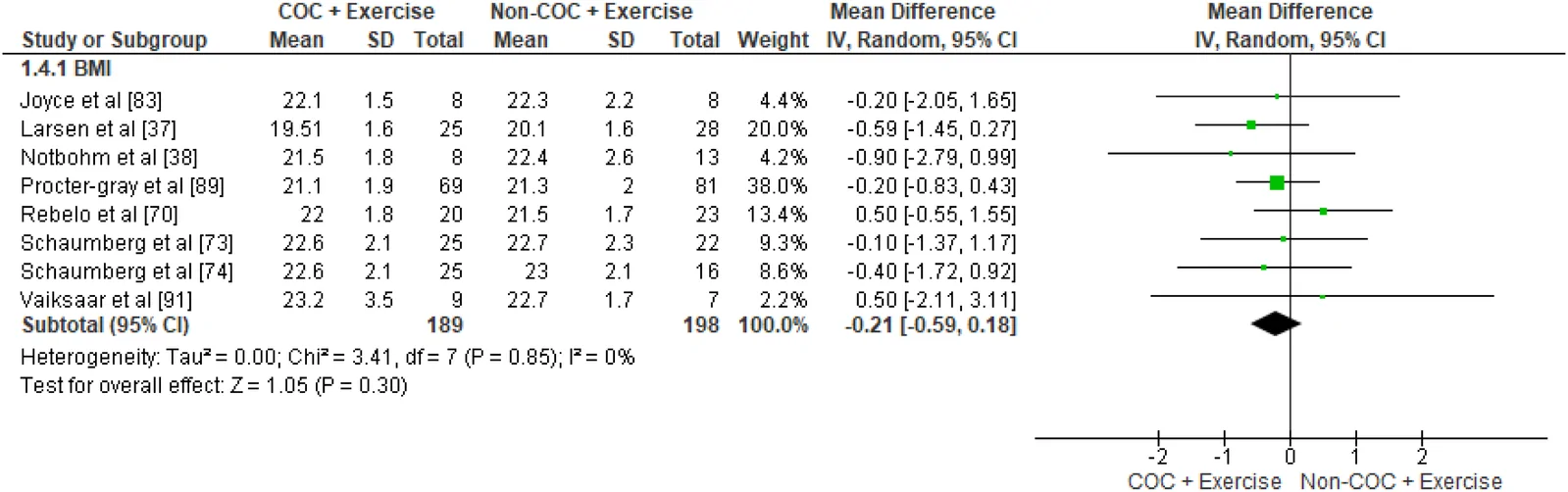
Forest plot showing the impact of COC on BMI in premenopausal women involved in exercise when compared with non-COC.

Similarly, the overall pooled estimate of the meta-analysis of studies with an independent comparator showed little to no difference in the BMI in those taking COC when compared to the non-COC group [MD = -0.21, 95% CI (−0.59, 0.18), *p* = 0.30 with a low level of heterogeneity (*I*^2^ = 0%, *p* = 0.85)] (low certainty evidence) (**Figure 6**). Thus, the result showed that COC use does not alter the BMI in premenopausal women engaged in exercise.

#### 3.5.2 Lipid profile

**Figure 7:**
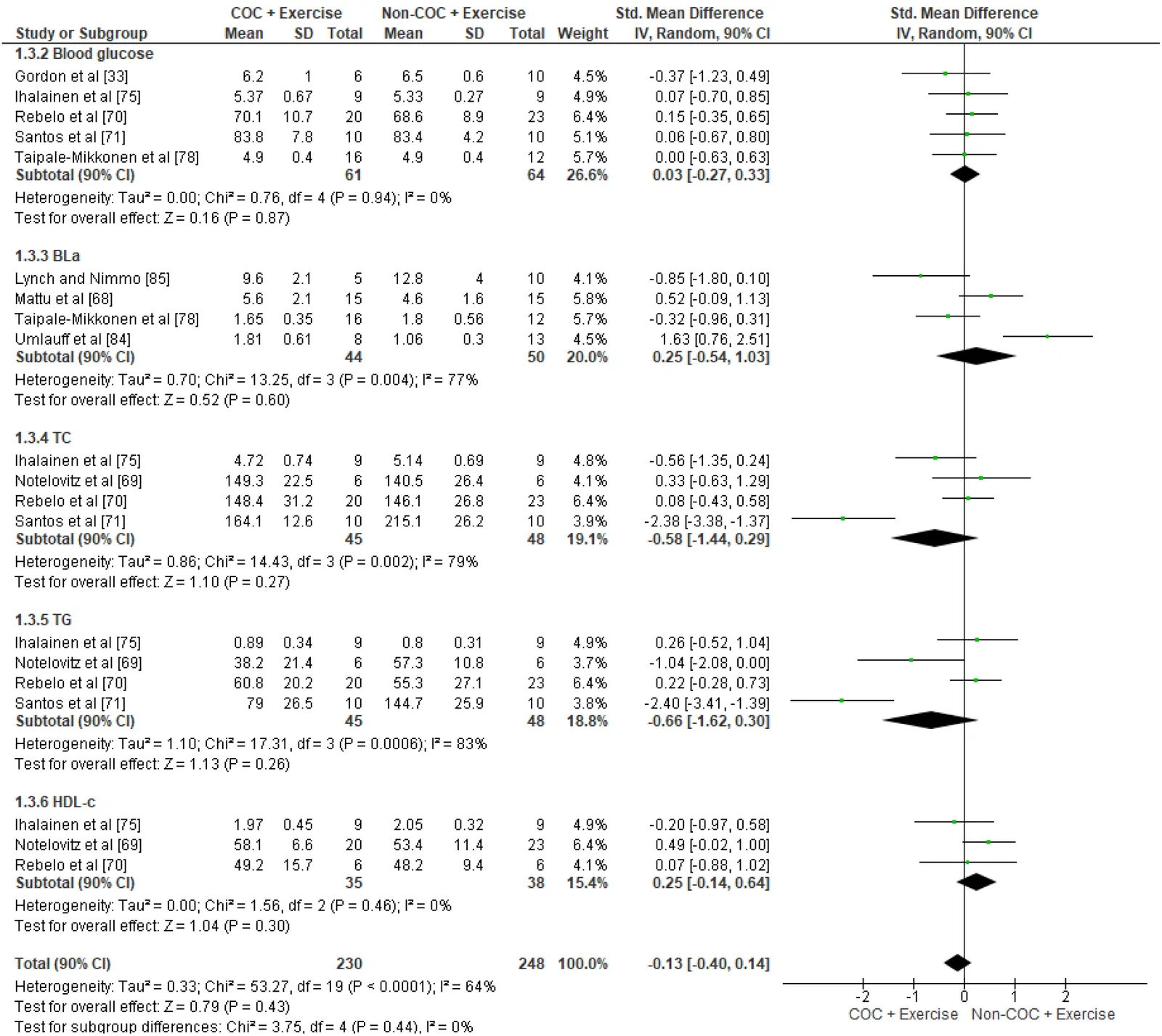
Forest plot showing the impact of COC on metabolic changes in premenopausal women engaged in exercise compared with non-COC. BLa, blood lactate; TC, total cholesterol; TG, triglyceride; HDL-c, high-density lipoprotein-cholesterol.

Overall, five studies reported the effect of COC on the lipid profile in premenopausal women engaged in exercise, as shown in **Table 1**. Three studies with an independent comparator showed that the impact of COC (ethinyl estradiol/progestin) within the dosage range of 15 µg–2 mg on the lipid profile of premenopausal women engaged in exercise is similar to that of the non-COC group (Notelovitz et al., 1987; Rebelo et al., 2010; Ihalainen et al., 2019). Similarly, evidence from two studies without an independent comparator showed that lipid profile levels remained unaltered in premenopausal women on COC and engaged in exercise (Lynch et al., 2001; Gomes et al., 2022). In contrast, two other studies with an independent comparator showed that COC (ethinyl estradiol/progestin) within the dosage range of 20 µg–3 mg increases the lipid profile levels (TC, LDL-C, and TG) in premenopausal women engaged in exercise compared with the non-COC group (Santos et al., 2008; Isacco et al., 2015). The overall pool estimates of TC in the meta-analysis of the studies with an independent comparator showed little to no difference in those taking COC when compared to the non-COC group with a moderate effect size [SMD = −0.58, 95% CI (−1.44, 0.29), *p* = 0.27, a high level of heterogeneity (*I*^2^ = 79%, *p* = 0.002)] (low certainty evidence) (**Figure 6**). For TG, the overall pool estimates of the meta-analysis of the studies with an independent comparator showed little to no difference in those taking COC when compared to the non-COC group with a moderate effect size [SMD = -0.66, 95% CI (−1.62, 0.30), *p* = 0.26, a high level of heterogeneity (*I*^2^ = 83%, *p* = 0.0006)] (low certainty evidence) (**Figure 6**), whereas for HDL the overall pool estimates of our meta-analysis of the studies with an independent comparator showed little to no difference in those taking COC when compared to non-COC group with a small effect size [SMD = 0.25, 95% CI (−0.14, 0.64), *p* = 0.30, a low level of heterogeneity (*I*^2^ = 0%, *p* = 0.46)] (low certainty evidence) (**Figure 7**). Thus, the result showed that COC use does not alter the lipid profile levels in premenopausal women engaged in exercise.

#### 3.5.3 Blood lactate

Overall, five studies reported the effect of COC on the blood lactate (BLa) levels in premenopausal women engaged in exercise, as shown in **Table 1**. Four studies with an independent comparator showed that the impact of COC (ethinylestradiol/progestin) within the dosage range of 15 µg–3 mg on BLa levels in premenopausal women engaged in exercise is similar to that of the non-COC group (Umlauff et al., 2021; Lynch and Nimmo, 1998; Mattu et al., 2020; Taipale-Mikkonen et al., 2021). In contrast, evidence from a cohort study without an independent comparator showed that monophasic COC within the dosage range of 20 µg–3 mg increased the mean BLa levels in premenopausal women engaged in exercise when compared with the inactive pill phase (Rechichi et al., 2008).

The overall pool estimates of BLa levels in the meta-analysis of the studies with an independent comparator showed little to no difference in those taking COC when compared to the non-COC group with a small effect size [SMD = 0.21, 95% CI (−0.61, 1.03), *p* = 0.68, a high level of heterogeneity (*I*^2^ = 79%, *p* = 0.002)] (low certainty evidence) (**Figure 7**). Thus, the result showed that COC use does not alter the BLa levels in premenopausal women engaged in exercise.

#### 3.5.4 Blood glucose

Overall, six studies reported the effect of COC on the blood glucose levels in premenopausal women engaged in exercise, as shown in **Table 1**. Five studies with an independent comparator showed that the impact of COC (ethinyl estradiol/progestin) within the dosage range of 15 µg–3 mg on blood glucose levels in premenopausal women engaged in exercise is similar to that of the non-COC group (Santos et al., 2008; Rebelo et al., 2010; Gordon et al., 2018; Ihalainen et al., 2019; Taipale-Mikkonen et al., 2021). Similarly, evidence from a cohort study without an independent comparator showed unaltered blood glucose levels across the phases of the pill cycle and inactive pill cycle in premenopausal women engaged in exercise (Lynch et al., 2001).

Similarly, the overall pool estimates of the blood glucose levels in the meta-analysis of studies with an independent comparator showed little to no difference in those taking COC when compared to the non-COC group with a small effect size [SMD = - 0.02, 95% CI (−0.32, 0.27), *p* = 0.89, a high level of heterogeneity (*I*^2^ = 0%, *p* = 0.88)] (low certainty evidence) (**Figure 7**). Thus, the result showed that COC use does not alter the blood glucose levels in premenopausal women engaged in exercise.

## 4 Discussion

The aim of this review was to provide a comprehensive synthesis of available evidence to evaluate the effect of COC on the markers of cardiorespiratory and immune function, as well as other health indicators, among recreationally active premenopausal women and female athletes engaged in exercise. The included studies in the review comprise 726 participants, of which 390 (53.7%) were taking OC and 336 (46.3%) were non-users, with a median age of 23 years. The outcome of this review indicates that COC is not associated with alterations in markers of cardiorespiratory function and IL-6 levels as well as other health indicators in premenopausal women compared with naturally menstruating women during exercise. Confidence in the pooled estimates is limited by the overall low certainty of the evidence, driven largely by the predominance of moderate-quality observational studies and the scarcity of randomized controlled trials. Methodological concerns, including small sample sizes, inadequate control of confounding factors, inconsistent reporting of COC formulations, and substantial heterogeneity in exercise protocols and outcome assessments, reduce the reliability and generalizability of the findings. Furthermore, pooling data from diverse COC formulations may mask clinically meaningful inter-individual responses, particularly among elite athletes and highly trained individuals, where even small physiological alterations may affect performance and recovery. Consequently, the findings should be interpreted cautiously, with greater weight given to evidence derived from longitudinal, crossover, and randomized study designs, which offer stronger control of bias and temporal variability than cross-sectional observational studies.

In the review, the effects of COC on specific cardiorespiratory outcomes are trivial and consistent across the included studies, reflecting a low inter-individual variability and a homogeneous group. The effect of COC on the participants’ HR during exercise did not yield meaningful changes compared with the non-COC group and was consistent with recent findings by Blake and colleagues (Blake et al., 2023). In contrast, evidence from a cohort study suggests an increase in HR during submaximal exercise in participants exposed to monophasic COC when compared to the naturally menstruating women in the non-COC group. The difference between the groups was accompanied by medium to large effect sizes (Taipale-Mikkonen et al., 2021). The suppressive effect of COC on endogenous sex hormone production underscores the role of reduced 17β-estradiol (E2) levels in promoting vasoconstriction and increased vascular resistance, which can subsequently contribute to elevated HR and blood pressure (Taipale-Mikkonen et al., 2021). However, a crossover study without an independent comparator group shows a lower resting HR in participants exposed to COC during exercise than in pre- and inactive exercise periods, and this is partly due to age and adaptation to high-intensity exercise, which help maintain cardiovascular function (Gomes et al., 2022). The lack of a standardized control group and of standardized exercise regimens contributes to this inconsistency in reported outcomes.

Furthermore, the between-group findings of the current review showed that COC did not significantly alter oxygen uptake, both at the submaximal (VO_2_) and maximal (VO_2_ max) levels, compared with the naturally menstruating women in the non-COC group during exercise, which is similar to recent findings where COC did not alter V̇O_2_ max among physically active women when compared with control groups (Schumpf et al., 2023). A pooled difference of less than 1 mL·kg^-1^·min^-1^ in VO_2_ max is generally considered within the normal day-to-day biological and technical variability of cardiopulmonary exercise testing, suggesting that the observed effects are unlikely to be clinically meaningful for most recreationally active women and female athletes. However, several studies showed a significant reduction in oxygen uptake among the participants that are exposed to COC during exercise when compared to naturally menstruating women in the non-COC group (Notelovitz et al., 1987; Lebrun et al., 2003; Joyce et al., 2013; Schaumberg et al., 2020). One study was a randomized, double-blind, placebo-controlled trial involving participants on the same triphasic COC for 2 months (Lebrun et al., 2003), while other studies were non-randomized and involved participants on various monophasic COC formulations for a longer duration (Notelovitz et al., 1987; Joyce et al., 2013; Schaumberg et al., 2020). Similarly, evidence from a longitudinal crossover study without an independent comparator group showed 13% reduction in VO_2_ after 4 months of low-dose triphasic COC when compared with baseline (Casazza et al., 2002). It is noteworthy that longitudinal intervention studies involving the same individuals, as well as randomized controlled trials, provide stronger evidence than cross-sectional observational studies. In the studies by Casazza et al. and Lebrun et al., the participants were exposed to the same type of COC, which potentially reduces inter-individual variation in endogenous sex hormones, thereby increasing the likelihood of a true effect (Casazza et al., 2002; Lebrun et al., 2003). This further supports the possibility of a decline in cardiorespiratory function in some individuals exposed to the COC compared with naturally menstruating women. In contrast, evidence from a longitudinal, randomized crossover design study suggests increased oxygen uptake among participants exposed to monophasic COC during exercise compared with baseline (Redman et al., 2005; Gomes et al., 2022). In these studies, the participants were exposed to COC for a longer period (6 months). In the study by Redman et al., the increased oxygen uptake in participants was attributed to the higher progestin dose (Rebelo et al., 2010), whereas in the study by Casazza et al. low-dose triphasic COC reduced VO_2_ max after 4 months (Casazza et al., 2002). In the crossover study by Gomes et al., increased oxygen uptake was associated with the cumulative effect of higher-intensity exercise compared with the period of inactivity in the participants (Gomes et al., 2022). Evidence from these studies underscores the functional role of high-endogenous progesterone (P4) secretion in adaptation to high-intensity exercise, helping maintain an elevated VO_2_ max to meet the metabolic demands of exercising muscle and enhancing exercise performance (Rael et al., 2021; Balakarthikeyan et al., 2023).

The interdependent relationship between ventilation (VE) and carbon dioxide production (VCO_2_), referred to as ventilatory efficiency (VE/VCO_2_), is considered a reliable predictor of prognosis and cardiorespiratory efficiency across various conditions (Guazzi et al., 2007; Schwaiblmair et al., 2012; Boutcher et al., 2013). The analysis in this review found that COC did not significantly affect VCO_2_ production or the VE levels in participants during exercise compared with naturally menstruating females in the non-COC groups. By contrast, evidence from longitudinal studies without independent comparators shows alterations in VE and VCO_2_ in participants exposed to COC (Casazza et al., 2002; Rechichi et al., 2008). In the longitudinal crossover study, participants exposed to the triphasic COC showed a reduction in VCO_2_ levels during exercise after 4 months from baseline. The participants were recreationally active individuals who received the same triphasic COC, resulting in a more homogeneous group and thereby reducing the influence of intra-individual differences on the reported outcome (Casazza et al., 2002). Unlike the longitudinal cohort study involving triathletes, exposure to different formulations of monophasic COC for a longer duration (>6 months) increased VE during exercise compared with the withdrawal phase. The exposure of athletes to different COC formulations reflected a heterogeneous population, highlighting the influence of substantial inter-individual variability in endogenous sex hormone profiles on the ventilatory responses (Rechichi et al., 2008). In exercise conditions, VE increases in proportion to metabolic demand (i.e., VCO_2_) to maintain arterial blood gas and acid–base balance (Davis et al., 2006).

Growing evidence highlights the importance of assessing the respiratory exchange ratio (RER), defined as the ratio of VCO_2_ to VO_2_, as an indicator of substrate utilization that reflects the relative contribution of carbohydrates and lipids to total energy expenditure under different exercise conditions (Ramos-Jiménez et al., 2008; Mezzani, 2017; Phillips et al., 2020). In this review, COC use did not significantly affect RER among participants engaged in exercise compared with the non-COC group. However, evidence from two randomized crossover trials indicates that COC significantly alters RER during exercise compared with the inactive pill phase (Giacomoni and Falgairette, 2000; Redman et al., 2005). In one study, a significant increase in RER during early exposure to COC was observed during submaximal steady-state exercise (Ihalainen et al., 2019). Furthermore, the participants were exposed to two formulations of monophasic COC for at least 18 months before entering the study, indicating a heterogeneous population (Ihalainen et al., 2019). In contrast, evidence from other studies showed that a higher progestin dose in COC decreased the RER levels during incremental exercise compared with a lower dose (Redman et al., 2005). The participants were exposed to the same COC formulation for 6 months, indicating a more homogeneous population and suggesting a dose-dependent effect of COC on RER (Redman et al., 2005). The exercise modalities, coupled with disparities in COC formulations and exposure durations, may account for the differences observed between the reported outcomes of these studies and those of the review. Evidence suggests that changes in exercise modality can activate distinct physiological pathways through which estrogen and/or progesterone exert their effects. While progesterone is known to modulate ventilatory drive, estrogen modulates substrate metabolism (Elliott-Sale et al., 2020). Estrogen can facilitate a switch from increased fatty acid oxidation to increased glycogen uptake and storage in the liver and muscles (Devries et al., 2006; Farinatti et al., 2016). While a high RER value in exercise conditions indicates predominant carbohydrate utilization, a low RER value suggests lipid oxidation (Frientes et al., 2023). Overall, the relatively consistent findings and low statistical heterogeneity across all cardiorespiratory outcomes strengthen confidence that COC use is unlikely to exert substantial, clinically meaningful effects on these parameters in most healthy premenopausal women when compared to non-users.

This review examined the effects of COC exposure on markers of immune function during exercise, and the findings suggest that COC use may influence specific aspects of exercise-induced immune responses, although the evidence remains inconsistent across studies. The immune system is essential for mediating exercise adaptations by maintaining the delicate balance between pro- and anti-inflammatory environments to preserve tissue homeostasis and promote physiological remodeling (Tidball and Wehling-Henricks, 2007; Deuster and Heled, 2008; Turner et al., 2016). A cohort study found that triphasic COC use was associated with greater exercise-induced cellular immune responses, including increases in total leukocytes, neutrophils, monocytes, and lymphocytes, compared with non-COC users, particularly during the luteal phase. Neutrophils demonstrated the most pronounced between-group effect, suggesting that synthetic sex hormones may augment neutrophil mobilization during exercise (Timmons et al., 2005).

In the cytokine milieu, the analysis of the review showed that COC did not significantly alter the IL-6 levels during exercise in participants taking COC compared with the non-COC group (Timmons et al., 2005; Ihalainen et al., 2019; Larsen et al., 2020; Notbohm et al., 2023b). However, substantial heterogeneity across studies can be attributed to differences in exercise intensity, training status, participant characteristics, and COC formulation. The duration and intensity of exercise are known contributory factors that can alter IL-6’s pleiotropic function (Nash et al., 2022).

Moreover, evidence from individual studies on other cytokines also suggests similar outcomes: IL-1ra, IL-8, IL-10, and TNF-alpha levels remained unaltered during exercise in participants exposed to COC, compared with the non-COC group (Larsen et al., 2020; Notbohm et al., 2023b). Contrastingly, other evidence showed that COC alters specific cytokine levels compared with the non-COC group (Bozzini et al., 2021; Notbohm et al., 2023b). A cohort study of recreationally active women found that COC decreased the IL-1β levels after exercise compared with the non-COC group (Notbohm et al., 2023b), whereas in a cross-sectional study of female collegiate athletes, IL-6 and TNF-α levels increased moderately in the post-season weeks of competitive events compared with their counterparts in the non-COC group. The observed increase in the COC group’s inflammatory response was attributed to their high training load (Bozzini et al., 2021). In the study by Notbohm et al. (2023b), the participants were exposed to various COC formulations and acute strength training, unlike the study by Bozzini et al. (2021) in which COC formulations were not reported and the participants engaged in more intense endurance and plyometric training exercise during competitive events.

Furthermore, evidence showed that COC had an effect on CRP levels during exercise compared with those without COC (Ihalainen et al., 2019; Larsen et al., 2020; Bozzini et al., 2021). CRP is regarded as an acute-phase reactant and maker of systemic inflammation (Paulsen et al., 2010). Evidence from cross-sectional studies showed higher CRP levels among athletes exposed to COC during exercise than in the non-COC group (Larsen et al., 2020; Bozzini et al., 2021). The participants in two of the studies were collegiate and Olympic athletes (Larsen et al., 2020; Bozzini et al., 2021), unlike in the study by Ihalainen et al., where the participants were recreationally active women (Ihalainen et al., 2019). Larsen et al. reported a threefold increase in athletes on COC compared to athletes without COC. The modality of exercise across the participants included strength and endurance exercise of high intensity (Ihalainen et al., 2019; Larsen et al., 2020; Bozzini et al., 2021), which is also similar to a crossover that showed increased CRP levels among participants who underwent high-intensity interval training when compared with baseline (Gomes et al., 2022). Variations in exercise modality, training intensity, heterogeneity in COC formulations, and varied methodological approaches to evaluating the measured outcomes across the included studies likely contributed to these divergent outcomes, as reflected in the high heterogeneity among the studies.

Notably, many components of the immune system express estrogen and progesterone receptors, highlighting their functional role in regulating the cytokine milieu during inflammatory responses (Singh et al., 2025). Emerging evidence has highlighted the modulatory role of estrogen on various immune cells in a dose-dependent manner, in which estrogen mechanistically inhibits nuclear factor kappa B (NF-κB) signaling through its receptors (Notbohm et al., 2023a). Low concentrations of estrogen can modulate the release of pro-inflammatory cytokines, whereas at high concentrations its modulatory effect can switch to stimulate the production of anti-inflammatory cytokines (Hirano et al., 2007; Singh et al., 2025).

Furthermore, exercise adaptation plays a crucial role in maintaining a delicate balance between pro- and anti-inflammatory environments, which may influence cardiorespiratory function—for instance, anti-inflammatory myokines, such as IL-10 and IL-6, released from skeletal muscle during moderate exercise, play a beneficial role in vasculature and muscle repair (Deng et al., 2012; Minuzzi et al., 2022). In contrast, pro-inflammatory TNF-alpha plays a deleterious role in muscle repair, promotes atherosclerotic processes, and impairs cardiac contractility and left ventricular remodeling, thereby diminishing cardiorespiratory function (Jønck et al., 2024). Evidence also suggests a significant correlation between decreased TNF-alpha levels and increased VO_2_ max following 3 months of endurance training (Smart et al., 2011). It is also noteworthy to highlight the role of immune responses and their association with metabolic adaptations that generally occur with regular exercise. Evidence from *in vitro* and *in vivo* studies shows that systemic IL-6 circulation promotes insulin sensitivity and glucose uptake via adenosine monophosphate-activated protein kinase (AMPK) activation in both skeletal muscle and adipose tissue during exercise (Larsen et al., 2002). Moreover, several studies have shown that IL-6 increases intramyocellular or whole-body fatty acid oxidation (Kelly et al., 2004; Glund et al., 2007). This evidence underscores the role of cytokines in perceived fatigue and the overtraining syndrome during exercise (Nash et al., 2022).

In this review, COC use did not significantly alter metabolic status or body composition in premenopausal women engaged in exercise when compared with naturally menstruating women or during the inactive pill phase. These findings are consistent with previous evidence reported by Horton and colleagues, who observed no significant changes in metabolic status across the menstrual cycle in moderately lean active women aged 18–39 years, either at rest or during prolonged moderate-intensity exercise (Petersen et al., 2005). The participant’s age and training regimen may account for the low heterogeneity and the trivial effect of COC on BMI (Horton et al., 2002). Despite the generally unchanged metabolic profile, some evidence indicated that COC use may influence specific metabolic markers during exercise. Alterations in lipid profile and blood lactate concentrations were observed among participants exposed to monophasic COC compared with non-users and during the inactive pill phase (Rechichi et al., 2008; Santos et al., 2008; Isacco et al., 2015)—for instance, the study by Isaaco and colleagues suggests that COC increased lipid peroxidation in women exposed to an incremental exercise intensity when compared with non-users (Isacco et al., 2015), which also corroborates findings by Santos et al. that reported higher levels of TC and TG among the participants when compared with the non-COC group (Santos et al., 2008). Similarly, a study by Rechichi and colleagues showed that COC increased the mean blood lactate level compared with the withdrawal phase in women engaged in exercise. The high variability in COC formulation and exercise modality may have contributed to the observed variations in metabolic changes across studies (Isacco et al., 2015).

It is worth noting that the strength of this review lies in its broad synthesis of available evidence on the impact of COC on cardiorespiratory and immune function across a wide range of participants, including recreationally active premenopausal women and trained female athletes across different geographical regions. The clinical relevance and sporting impact of our review also extend to the periodization of exercise among recreationally active and athletic women, given fluctuations in hormonal secretion. Nonetheless, this review presents several drawbacks, including a limited number of randomized controlled trials, variations in the type and intensity of exercise among participants, variability in the brand and formulations of COC, a small sample size, and varied methodological approaches to evaluating the measured outcomes across the included studies. The classification of participants as recreationally active, trained, or elite, as well as their training status, influences the magnitude and interpretation of physiological responses and limits the generalizability of our findings. Recreationally active individuals generally demonstrate broad adaptations associated with health-related fitness, whereas trained athletes exhibit sport-specific adaptations resulting from systematic training. Elite athletes represent a highly adapted and selectively developed population, where physiological responses may reflect optimization near biological limits. Therefore, findings derived from one population should be interpreted cautiously when extrapolated across different levels of athletic development. Moreover, the lack of data from certain geographical locations is also a drawback for generalizing our findings. Therefore, there is a need for more rigorously designed longitudinal and randomized studies incorporating standardized comparators, homogeneous populations, larger sample sizes, and improved methodological approaches. Such studies, alongside more cost-effective research strategies, would help clarify the true effects of COC exposure on the reported outcomes among women of reproductive age and beyond. Furthermore, future research should comprehensively report the specific COC formulations administered to participants to facilitate reproducibility and appropriate subgroup classification, given that variations in COC composition may differentially influence endogenous hormonal profiles and contribute to between-participant variability. This will enhance decision-making regarding the safety profiles of available COCs in and out of clinical settings.

## 5 Conclusion

Overall, this current review suggests no consistent adverse effect of COC use on cardiorespiratory or immune outcomes among physically active premenopausal women. However, confidence in these findings remains limited due to the low certainty of available evidence. This can be attributed to the methodological concern across the included studies, such as selection bias, variability in participants and the brand and formulation of COC, a relatively small sample size of participants, the varied methodological approaches to evaluate the outcomes by all the included studies, and a lack of sufficient evidence in other geographical locations, among other factors. Thus, caution should be exercised when interpreting and extrapolating these findings, especially during the decision-making process to select from the available COCs.

## Data Availability

The original contributions presented in the study are included in the article/**Supplementary Material**. Further inquiries can be directed to the corresponding author.
