# Correction: Effect of oral contraceptive use on cardiorespiratory and immune function in premenopausal women involved in exercise: a systematic review and meta-analysis

**DOI:** 10.3389/fphys.2026.1951039

**Published:** 2026-09-02

**Authors:** Oyesanmi A. Fabunmi, Gugulethu P. Luthuli, Phiwayinkosi V. Dludla, Bongani B. Nkambule

**Affiliations:** 1 Section Sports Medicine, Faculty of Health Sciences, University of Pretoria, Pretoria, South Africa; 2 Health-awareness, Exercise and Cardio-immunologic Research Unit (HECIRU), Department of Physiology, College of Medicine, Ekiti State University, Ado-Ekiti, Ekiti State, Nigeria; 3 Sport Exercise Medicine and Lifestyle Institute (SEMLI), Faculty of Health Sciences, University of Pretoria, Pretoria, South Africa; 4 School of Medicine, College of Health Sciences, University of KwaZulu-Natal, Durban, South Africa; 5 Department of Biochemistry and Microbiology, University of Zululand, KwaDlangezwa, South Africa

**Keywords:** cardiorespiratory function, combined oral contraceptive, exercise, geographical location, immune function, luteal phase, premenopausal women

A correction has been made to the section **2 Methods**, “2.1 *Eligibility criteria*”. “Studies reporting on non-smokers were excluded” was incorrect. The corrected sentence is:

“Studies reporting on smokers were excluded from our review because smoking is regarded as an unhealthy lifestyle, which contradicts the focus of our study regarding healthy participants”.

The original version of this article has been updated.

