# Supplementary material for: Effect of oral contraceptive use on cardiorespiratory and immune function in premenopausal women involved in exercise: a systematic review and meta-analysis
Source: Front Physiol. 2026 Jul 22;17:1728032. doi: 10.3389/fphys.2026.1728032 (PMC13437507; doi:10.3389/fphys.2026.1728032)
Supplement: Supplementary file 1 [file Table1.doc]

**Supplementary file.**

**Table 2:** search strategy

| Search | Query | Records retrieved |
| --- | --- | --- |
| #1 | ("contraceptives, oral"[MeSH Terms] OR ("contraceptives"[All Fields] AND "oral"[All Fields]) OR "oral contraceptives"[All Fields] OR ("oral"[All Fields] AND "contraceptives"[All Fields]) OR ("contraception"[MeSH Terms] OR "contraception"[All Fields] OR ("birth"[All Fields] AND "control"[All Fields]) OR "birth control"[All Fields]) OR ("contraceptives oral combined"[Pharmacological Action] OR "contraceptives, oral, combined"[MeSH Terms] OR ("contraceptives"[All Fields] AND "oral"[All Fields] AND "combined"[All Fields]) OR "combined oral contraceptives"[All Fields] OR ("combined"[All Fields] AND "oral"[All Fields] AND "contraceptives"[All Fields])) OR (("contraceptives, oral"[MeSH Terms] OR ("contraceptives"[All Fields] AND "oral"[All Fields]) OR "oral contraceptives"[All Fields] OR ("oral"[All Fields] AND "contraceptive"[All Fields]) OR "oral contraceptive"[All Fields]) AND ("pill s"[All Fields] OR "pills"[All Fields])) OR ("contracept"[All Fields] OR "contracepted"[All Fields] OR "contracepting"[All Fields] OR "contraception"[MeSH Terms] OR "contraception"[All Fields] OR "contraceptions"[All Fields] OR "contraceptive agents"[Pharmacological Action] OR "contraceptive agents"[MeSH Terms] OR ("contraceptive"[All Fields] AND "agents"[All Fields]) OR "contraceptive agents"[All Fields] OR "contraceptives"[All Fields] OR "contraceptive devices"[MeSH Terms] OR ("contraceptive"[All Fields] AND "devices"[All Fields]) OR "contraceptive devices"[All Fields] OR "contraceptive"[All Fields] OR "contraceptive s"[All Fields] OR "contraceptively"[All Fields])) AND (fft[Filter]) | 178,583 |
| #2 | "exercise"[MeSH Terms] OR "exercise"[All Fields] OR "exercises"[All Fields] OR "exercise therapy"[MeSH Terms] OR ("exercise"[All Fields] AND "therapy"[All Fields]) OR "exercise therapy"[All Fields] OR "exercise s"[All Fields] OR "exercised"[All Fields] OR "exerciser"[All Fields] OR "exercisers"[All Fields] OR "exercising"[All Fields] OR ("physical fitness"[MeSH Terms] OR ("physical"[All Fields] AND "fitness"[All Fields]) OR "physical fitness"[All Fields]) OR ("athlete s"[All Fields] OR "athletes"[MeSH Terms] OR "athletes"[All Fields] OR "athlete"[All Fields] OR "athletically"[All Fields] OR "athlets"[All Fields] OR "sports"[MeSH Terms] OR "sports"[All Fields] OR "athletic"[All Fields] OR "athletics"[All Fields]) OR (("heart"[MeSH Terms] OR "heart"[All Fields] OR "cardio"[All Fields]) AND ("workout"[All Fields] OR "workouts"[All Fields])) | 804,474 |
| #3 | "Cardiorespiratory fitness"[MeSH Terms] OR ("cardiorespiratory"[All Fields] AND "fitness"[All Fields]) OR "cardiorespiratory fitness"[All Fields] | 9,857 |
| #3 | ("immune"[All Fields] OR "immuned"[All Fields] OR "immunes"[All Fields] OR "immunisation"[All Fields] OR "vaccination"[MeSH Terms] OR "vaccination"[All Fields] OR "immunization"[All Fields] OR "immunization"[MeSH Terms] OR "immunisations"[All Fields] OR "immunizations"[All Fields] OR "immunise"[All Fields] OR "immunised"[All Fields] OR "immuniser"[All Fields] OR "immunisers"[All Fields] OR "immunising"[All Fields] OR "immunities"[All Fields] OR "immunity"[MeSH Terms] OR "immunity"[All Fields] OR "immunization s"[All Fields] OR "immunize"[All Fields] OR "immunized"[All Fields] OR "immunizer"[All Fields] OR "immunizers"[All Fields] OR "immunizes"[All Fields] OR "immunizing"[All Fields]) AND ("activable"[All Fields] OR "activate"[All Fields] OR "activated"[All Fields] OR "activates"[All Fields] OR "activating"[All Fields] OR "activation"[All Fields] OR "activations"[All Fields] OR "activator"[All Fields] OR "activator s"[All Fields] OR "activators"[All Fields] OR "active"[All Fields] OR "actived"[All Fields] OR "actively"[All Fields] OR "actives"[All Fields] OR "activities"[All Fields] OR "activity s"[All Fields] OR "activitys"[All Fields] OR "motor activity"[MeSH Terms] OR ("motor"[All Fields] AND "activity"[All Fields]) OR "motor activity"[All Fields] OR "activity"[All Fields]) | 569,901 |
| #4 | ("Pre-menopausal"[All Fields] AND ("womans"[All Fields] OR "women"[MeSH Terms] OR "women"[All Fields] OR "woman"[All Fields] OR "women s"[All Fields] OR "womens"[All Fields])) OR (("young"[All Fields] OR "youngs"[All Fields]) AND ("femal"[All Fields] OR "female"[MeSH Terms] OR "female"[All Fields] OR "females"[All Fields] OR "female s"[All Fields] OR "femals"[All Fields]) AND ("adult"[MeSH Terms] OR "adult"[All Fields] OR "adults"[All Fields] OR "adult s"[All Fields])) | 1,023,985 |
| #5 | #1 AND #2 OR #3AND #4  Combination of strings 1, 2, 3 and 4 using the Boolean term “OR”, “AND” | 351 |

The keywords and MeSH terms used included “oral contraceptive pills”, “birth control pills’’, “oral contraceptives”, “contraceptives”, “exercise”, “physical activity” “cardiorespiratory fitness”, “immune activation”, and “premenopausal women”. There are no database search date limits and no language limits.

|  | **Table 3. Quality Appraisal of included studies** | | |  |  |  |  |  |  |  |  |  |  |  |  |  |  |
| --- | --- | --- | --- | --- | --- | --- | --- | --- | --- | --- | --- | --- | --- | --- | --- | --- | --- |
| ***JBI Critical Appraisal Checklist***  ***for Randomized Controlled Studies*** | **Author (s) / year** | **Q1** | **Q2** | **Q3** | **Q4** | **Q5** | **Q6** | **Q7** | **Q8** | **Q9** | **Q10** | **Q11** | **Q12** | **Q13** | **Quality score** | **Kappa score** |  |
|  | Giacomoni and falgairette / 2000 | 0 | 0 | 1 | 0 | 0 | 0 | 1 | 1 | 1 | 1 | 1 | 1 | 1 | 8/13 | 0.69 |  |
|  | Lebrun et al / 2003 | 0 | 0 | 1 | 1 | 1 | 0 | 1 | 1 | 1 | 1 | 1 | 1 | 1 | 11/13 | 0.38 |  |
|  | Redman et al / 2005 | 0 | 0 | 0 | 1 | 0 | 0 | 1 | 1 | 1 | 1 | 1 | 1 | 1 | 8/13 | 0.69 |  |
|  | Procter-gray et al / 2008 | 1 | 0 | 1 | 0 | 0 | 1 | 1 | 1 | 1 | 1 | 1 | 0 | 0 | 8/13 | 0.69 |  |
|  |  |  |  |  |  |  |  |  |  |  |  |  |  |  |  |  |  |
|  | ***Mean average*** |  |  |  |  |  |  |  |  |  |  |  |  |  | **8.8/13** | **0.61** |  |
|  | ***lower C.I*** |  |  |  |  |  |  |  |  |  |  |  |  |  |  | **0.37** |  |
|  | ***Upper CI*** |  |  |  |  |  |  |  |  |  |  |  |  |  |  | **0.86** |  |
|  |  |  |  |  |  |  |  |  |  |  |  |  |  |  |  |  |  |
| ***JBI Critical Appraisal Checklist for Cohort Studies*** | Taipale-Mikkonen et al / 2021 | 1 | 1 | 1 | 1 | 1 | 1 | 1 | 1 | 1 | 0 | 1 |  |  | 10/11 | 0.64 |  |
|  | Casazza et al / 2002 | 1 | 1 | 1 | 1 | 0 | 1 | 1 | 0 | 1 | 0 | 1 |  |  | 8/11 | 0.64 |  |
|  | Vaiksaar et al / 2011 (a) Phase | 1 | 1 | 1 | 0 | 0 | 1 | 1 | 1 | 1 | 1 | 1 |  |  | 9/11 | 0.64 |  |
|  | Vaiksaar et al / 2011 (b) | 1 | 1 | 1 | 1 | 1 | 1 | 1 | 1 | 1 | 1 | 1 |  |  | 11/11 | 0.45 |  |
|  | Rechichi et al / 2008 | 1 | 1 | 1 | 1 | 0 | 0 | 1 | 1 | 1 | 1 | 1 |  |  | 9/11 | 0.27 |  |
|  | Timmons et al / 2005 | 0 | 0 | 0 | 1 | 1 | 0 | 1 | 1 | 1 | 1 | 1 |  |  | 7/11 | 0.09 |  |
|  | Lynch et al / 2001 | 1 | 1 | 0 | 1 | 1 | 0 | 1 | 1 | 1 | 1 | 1 |  |  | 9/11 | 0.09 |  |
|  | Isacco et al / 2014 | 1 | 1 | 1 | 1 | 1 | 1 | 1 | 1 | 1 | 1 | 1 |  |  | 11/11 | 0.45 |  |
|  | Bryner et al / 1996 | 0 | 1 | 1 | 0 | 0 | 1 | 1 | 1 | 1 | 1 | 1 |  |  | 8/11 | 0.45 |  |
|  | Joyce et al / 2013 | 1 | 1 | 1 | 1 | 1 | 1 | 1 | 1 | 1 | 1 | 1 |  |  | 11/11 | 0.27 |  |
|  | Notbohm et al / 2023 | 1 | 1 | 1 | 1 | 1 | 0 | 1 | 1 | 1 | 1 | 1 |  |  | 10/11 | 0.82 |  |
|  | Umlauff et al/ 2021 | 1 | 1 | 1 | 1 | 1 | 1 | 1 | 1 | 1 | 1 | 1 |  |  | 11/11 | 0.82 |  |
|  | Lynch and Nimmo / 1998 | 1 | 1 | 1 | 1 | 1 | 1 | 0 | 1 | 0 | 1 | 1 |  |  | 9/11 | 0.45 |  |
|  |  |  |  |  |  |  |  |  |  |  |  |  |  |  |  |  |  |
|  | ***Mean average*** |  |  |  |  |  |  |  |  |  |  |  |  |  | **9.4/11** | **0.51** |  |
|  | ***lower C.I*** |  |  |  |  |  |  |  |  |  |  |  |  |  |  | **0.35** |  |
|  | ***Upper CI*** |  |  |  |  |  |  |  |  |  |  |  |  |  |  | **0.66** |  |
|  |  |  |  |  |  |  |  |  |  |  |  |  |  |  |  |  |  |
| ***JBI Critical Appraisal Checklist for Cross-sectional Studies*** | Mattu et al / 2020 | 1 | 1 | 1 | 1 | 1 | 1 | 1 | 1 |  |  |  |  |  | 8/8 | 0.5 |  |
|  | Larsen et al / 2020 | 0 | 1 | 1 | 1 | 0 | 0 | 1 | 1 |  |  |  |  |  | 5/8 | 0.75 |  |
|  | Notelovitz et al / 1987 | 1 | 1 | 0 | 1 | 0 | 0 | 1 | 1 |  |  |  |  |  | 5/8 | 0.25 |  |
|  | Rebelo et al / 2009 | 1 | 1 | 0 | 1 | 1 | 0 | 1 | 1 |  |  |  |  |  | 5/8 | 0.25 |  |
|  | Santos et al / 2008 | 1 | 1 | 0 | 1 | 0 | 0 | 1 | 1 |  |  |  |  |  | 5/8 | 0.25 |  |
|  | Gomes et al / 2022 | 1 | 1 | 1 | 1 | 1 | 0 | 1 | 1 |  |  |  |  |  | 7/8 | 0.75 |  |
|  | Schaumberg et al / 2020 | 1 | 1 | 1 | 1 | 0 | 0 | 1 | 1 |  |  |  |  |  | 6/8 | 0.5 |  |
|  | Schaumberg et al / 2017 | 1 | 1 | 1 | 1 | 0 | 0 | 1 | 1 |  |  |  |  |  | 6/8 | 1 |  |
|  | Ihalainen et al / 2019 | 1 | 1 | 1 | 1 | 1 | 1 | 1 | 1 |  |  |  |  |  | 8/8 | 0.5 |  |
|  | Bozzini et al / 2021 | 1 | 1 | 1 | 1 | 1 | 1 | 1 | 1 |  |  |  |  |  | 8/8 | 1 |  |
|  | Nakamura and Nose-Ogura / 2021 | 1 | 1 | 1 | 1 | 1 | 0 | 1 | 1 |  |  |  |  |  | 7/8 | 1 |  |
|  |  |  |  |  |  |  |  |  |  |  |  |  |  |  |  |  |  |
|  | ***Mean average*** |  |  |  |  |  |  |  |  |  |  |  |  |  | **6.4/8** | **0.61** |  |
|  | ***lower C.I*** |  |  |  |  |  |  |  |  |  |  |  |  |  |  | **0.41** |  |
|  | ***Upper CI*** |  |  |  |  |  |  |  |  |  |  |  |  |  |  | **0.82** |  |
|  |  |  |  |  |  |  |  |  |  |  |  |  |  |  |  |  |  |
| ***KEY****: 1 = Yes; 0 = No* |  |  |  |  |  |  |  |  |  |  |  |  |  |  |  |  |  |

A.

B.

**Figure 6A-C**: Funnel plot of cardiovascular hemodynamic (Heart rate), respiratory changes, metabolic and body composition of participants involved in exercise.

**Table 4. Summary of findings:** Effect of oral contraceptive use on cardiorespiratory function and immune activation in premenopausal women involved in exercise.

| COC + Exercise Versus Non-COC + Exercise for Premenopausal Women | | | | | | |  |
| --- | --- | --- | --- | --- | --- | --- | --- |
| Outcome | Patients (studies), N | Relative effect (95% CI) | **Absolute effects (95% CI)** | | | Certainty | What happens |
|  |  |  | **Non-COC + Exercise** | **COC + Exercise** | **Difference** |  |  |
| **Cardiovascular hemodynamic** |  |  | | | |  |  |
| Heart rate | 288 (11 studies) | - |  | - | **MD**  **0.02** (-1.85 to 1.9) | ⨁⨁◯◯ Low^a,b^ | The evidence suggests that COC + Exercise results in little to no difference in heart rate. |
| **Respiratory changes** |  |  | | | |  |  |
| VO_2_ | 203 (8 studies) | - | - | - | **SMD**  **-0.24** (-0.48 to 0) | ⨁⨁◯◯ Low^a,b^ | The evidence suggests that COC + Exercise results in little to no difference in VO2 . |
| VO_2_ max | 248 (12 studies) | - | - | - | **SMD**  **0.02** (-0.2 to 0.23) | ⨁⨁◯◯ Low^a,b^ | The evidence suggests that COC + Exercise results in little to no difference in VO2 max. |
| VCO_2_ | 130 (5 studies) | - | - | - | **SMD**  **-0.21** (-0.55 to 0.13) | ⨁⨁◯◯ Low^a,b^ | The evidence suggests that COC + Exercise results in little to no difference in VCO2. |
| RER | 176 (8 studies) | - | - | - | **SMD**  **0.07** (-0.018 to 0.32) | ⨁⨁◯◯ Low^a,b^ | The evidence suggests that COC + Exercise results in little to no difference in RER. |
| VE | 188 (9 studies) | - | - | - | **SMD**  **-0.07** (-0.39 to 0.25) | ⨁⨁◯◯ Low^a,b^ | The evidence suggests that COC + Exercise results in little to no difference in VE. |
| **Markers of immune activation** | 139 (6 studies) | The outcome of our study demonstrated differential changes in the immune cell count as well as inflammatory markers such as CRP, IL-6, IL-1β, TNF-α, and IL-8. | | | | ⨁⨁◯◯ Low^a,c^ | COC + Exercise may result in a slight change in markers of immune activation. |
| **Body composition and metabolic changes** |  |  | | | |  |  |
| BMI | 387 (8 studies) | - | - | - | **MD**  **-0.21** (-0.59 to 0.18) | ⨁⨁◯◯ Low^a,b^ | The evidence suggests that COC + Exercise results in little to no difference in BMI. |
| Blood glucose | 125 (5 studies) | - | - | - | **SMD**  **-0.02** (-0.32 to 0.27) | ⨁⨁◯◯ Low^a,b^ | The evidence suggests that COC + Exercise results in little to no difference in blood glucose. |
| BLa | 94 (4 studies) | - | - | - | **SMD**  **0.21** (-0.61 to 1.03) | ⨁⨁◯◯ Low^a,b^ | The evidence suggests that COC + Exercise results in little to no difference in bLa. |
| TC | 93 (4 studies) | - | - | - | **SMD**  **-0.58** (-1.44 to 0.29) | ⨁⨁◯◯ Low^a,b^ | The evidence suggests that COC + Exercise results in little to no difference in TC . |
| TG | 93 (4 studies) | - | - | - | **SMD**  **-0.66** (-1.62 to 0.32) | ⨁⨁◯◯ Low^a,b^ | The evidence suggests that COC + Exercise results in little to no difference in TG. |
| HDL-C | 73 (3 studies) | - |  | - | **SMD**  **0.25** (-0.14 to 0.64) | ⨁⨁◯◯ Low^a,b^ | The evidence suggests that COC + Exercise results in little to no difference in HDL-C . |
| **CI:** confidence interval; **MD:** mean difference; **SMD:** standardised mean difference | | | | | | |  |

a. lack of participants and assessor blinding as well as lack of allocation concealment

b. The sample size of each outcome is quite small to meet the threshold for optimal information size.

c. There are differences in how the interventions are administered in terms of duration and dosage of COC as well as the time frame of outcome measurement.
